# Mitochondrial pyruvate metabolism and glutaminolysis toggle steady-state and emergency myelopoiesis

**DOI:** 10.1084/jem.20221373

**Published:** 2023-05-30

**Authors:** Hannah A. Pizzato, Yahui Wang, Michael J. Wolfgang, Brian N. Finck, Gary J. Patti, Deepta Bhattacharya

**Affiliations:** 1 Department of Pathology and Immunology, Washington University School of Medicine, Saint Louis, MO, USA; 2 Department of Immunobiology, University of Arizona, Tucson, AZ, USA; 3 Department of Chemistry, Washington University, Saint Louis, MO, USA; 4 Department of Biological Chemistry, Johns Hopkins University School of Medicine, Baltimore, MD, USA; 5 Department of Pharmacology and Molecular Sciences, Johns Hopkins University School of Medicine, Baltimore, MD, USA; 6 Division of Geriatrics and Nutritional Sciences, Washington University School of Medicine, Saint Louis, MO, USA; 7 Department of Medicine, Washington University School of Medicine, Saint Louis, MO, USA; 8 Siteman Cancer Center, Washington University, Saint Louis, MO, USA; 9 BIO5 Institute, University of Arizona, Tucson, AZ, USA

## Abstract

To define the metabolic requirements of hematopoiesis, we examined blood lineages in mice conditionally deficient in genes required for long-chain fatty acid oxidation (*Cpt2*), glutaminolysis (*Gls*), or mitochondrial pyruvate import (*Mpc2*). Genetic ablation of *Cpt2* or *Gls* minimally impacted most blood lineages. In contrast, deletion of *Mpc2* led to a sharp decline in mature myeloid cells and a slower reduction in T cells, whereas other hematopoietic lineages were unaffected. Yet MPC2-deficient monocytes and neutrophils rapidly recovered due to a transient and specific increase in myeloid progenitor proliferation. Competitive bone marrow chimera and stable isotope tracing experiments demonstrated that this proliferative burst was progenitor intrinsic and accompanied by a metabolic switch to glutaminolysis. Myeloid recovery after loss of MPC2 or cyclophosphamide treatment was delayed in the absence of GLS. Reciprocally, MPC2 was not required for myeloid recovery after cyclophosphamide treatment. Thus, mitochondrial pyruvate metabolism maintains myelopoiesis under steady-state conditions, while glutaminolysis in progenitors promotes emergency myelopoiesis.

## Teaser

Steady-state and emergency myelopoiesis require different rates of mature cell production to maintain or restore homeostasis. This study demonstrates that mitochondrial pyruvate is specifically required for steady-state but not emergency myelopoiesis. Reciprocally, glutaminolysis specifically promotes emergency but not steady-state myelopoiesis.

## Graphical Abstract

**Figure:**
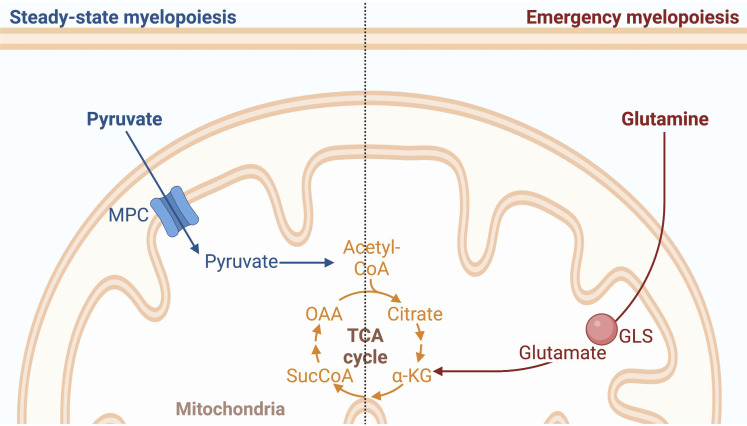

## Introduction

As hematopoietic stem cells (HSCs) differentiate, they gradually lose the potential to generate specific blood cell types until the commitment to a single lineage is achieved. Since HSCs are exceedingly rare, their differentiation occurs concomitantly with a progressive expansion of downstream progenitors to ensure sufficient production of mature cells (Bryder et al., 2006). Along with a numerical expansion, downstream progenitors possess both more proliferative and differentiative capacity than HSCs (Passegué et al., 2005; Sun et al., 2014). This progressive expansion of progenitors and mature lineages is known as transit amplification. Under homeostatic conditions, transit amplification matches the rate of new cell generation with the turnover of mature cells. Because each mature lineage has its own distinct lifespan, the mechanisms that maintain homeostasis are necessarily complicated. For example, neutrophils do not survive for more than a few days, while mature B lymphocytes persist for several months (Cronkite et al., 1959; Fulcher and Basten, 1997; Mauer et al., 1960; Pillay et al., 2010). The different arms of hematopoiesis must therefore be controlled in modular and separable ways.

Feedback triggered by an acute loss of downstream cells to upstream progenitors may require a distinct response to restore homeostasis. As one example, repeated bleeding of mice triggers HSCs to proliferate and self-renew at an increased rate, leading to the restoration of bone marrow cells and increased splenocytes (Cheshier et al., 2007). Upon transfusion of RBCs in these mice, HSC proliferation returns to normal. As another example, transplantation of a small number of WT HSCs into RAG2 and IL-2 receptor common γ chain–deficient mice, which lack B, T, and natural killer (NK) cells, results in a rapid repopulation of mature lymphocytes (Bhattacharya et al., 2006). This is driven by a pronounced and selective expansion of B and T cell progenitors despite very low levels of HSC chimerism. These data suggest that HSCs and progenitors respond to fill a void left by the loss of more differentiated downstream progenitors and mature cells in a process called emergency hematopoiesis. The intrinsic mechanisms that allow cells to shift from steady-state to emergency hematopoiesis still remain to be defined (Venezia et al., 2004).

Metabolic adaption might permit hematopoietic progenitors to adjust output in response to demand. For example, a decrease in lymphocytes occurs within hours of streptozotocin treatment (Muller et al., 2011), which ablates pancreatic beta cells and induces diabetes and high glucose levels (Rakieten et al., 1963; Arison et al., 1967; Lenzen, 2008). Yet by 4 wk after treatment with streptozotocin, lymphocyte numbers return to normal (Nagareddy et al., 2013). Similarly, mouse lymphocytes decrease after 6 wk on a high-fat diet (Luo et al., 2015) but increase after 90 d (Trottier et al., 2012). Moreover, nutrient availability varies substantially throughout the course of the day (Pickel and Sung, 2020; Potter et al., 2016; Schlierf and Dorow, 1973), yet there is relatively little impact on the abundances of mature cells (Born et al., 1997; Haus et al., 1983; Lawrence et al., 1933; Sabin et al., 1927). These examples represent independent studies and are not directly comparable to each other. Nonetheless, the data hint that while severe metabolic alterations may transiently alter hematopoietic composition, progenitors may be able to adapt to these changes to recover and maintain homeostasis. Defining whether and how such an adaptation occurs requires genetic tools and a dissection of progenitor-intrinsic metabolic pathways.

Much of the work performed to date on hematopoietic metabolism has focused on the relative dependence on glycolysis versus mitochondrial functions by HSCs and progenitors. The metabolic profile of HSCs is notably different than those of lineage-committed progenitors (Agathocleous et al., 2017). HSCs generally rely on glycolysis to remain quiescent, but as differentiation proceeds, a shift to increased oxidative phosphorylation occurs to ensure bioenergetic requirements are met (Takubo et al., 2013; Yu et al., 2013; Wang et al., 2014; Maryanovich et al., 2015; Ito and Suda, 2014; Nakamura-Ishizu et al., 2020; Simsek et al., 2010; Suda et al., 2011; Ho et al., 2017). Accordingly, lineage-committed progenitors produce more reactive oxygen species than do upstream HSCs, and increasing levels of reactive oxygen species hinders HSC function (Inoue et al., 2010; Ito et al., 2004; Ito et al., 2006; Norddahl et al., 2011; Tan and Suda, 2018; Tothova et al., 2007; Wang et al., 2014). Though HSCs do still rely on mitochondrial functions (Ansó et al., 2017; Bejarano-García et al., 2016; Chen et al., 2008; Gan et al., 2010; Gurumurthy et al., 2010; Luchsinger et al., 2016; Maryanovich et al., 2012; Mortensen et al., 2011; Nakada et al., 2010; Qi et al., 2021; Umemoto et al., 2018; Vannini et al., 2016), the preponderance of evidence suggests that hematopoietic progenitors depend more heavily on oxidative phosphorylation of carbon sources to generate ATP. The specific carbon sources that fuel oxidative phosphorylation in hematopoietic progenitors in vivo have not been genetically defined, though in a broad sense glucose is essential for M-CSF–driven myelopoiesis (Karmaus et al., 2017). Assuming distinct bioenergetic requirements, it is possible that each blood lineage preferentially relies on different carbon sources and metabolic pathways.

Here, we utilized in vivo genetic models to interrogate the roles of long-chain fatty acid oxidation, glutaminolysis, and mitochondrial pyruvate import in hematopoietic homeostasis. In a striking example of homeostatic maintenance, myeloid progenitors genetically deprived of mitochondrial pyruvate not only switched to glutaminolysis, but this switch was also accompanied by a transient and rapid proliferative burst to regenerate only themselves and their mature myeloid daughters, whereas other progenitors and lineages were unaffected. While glutaminolysis is not required for the homeostatic maintenance of myeloid cells, it is generally required for emergency myelopoiesis even when mitochondrial pyruvate metabolism is available. These data demonstrate that progenitors have intrinsic metabolic sensing abilities that promote lineage-specific hematopoietic homeostasis. Additionally, the metabolic pathways utilized to maintain myeloid cells under steady-state differ from those used under situations of acute insult.

## Results

### Long-chain fatty acid oxidation and glutaminolysis are dispensable for most hematopoietic lineages

**Figure 1.**
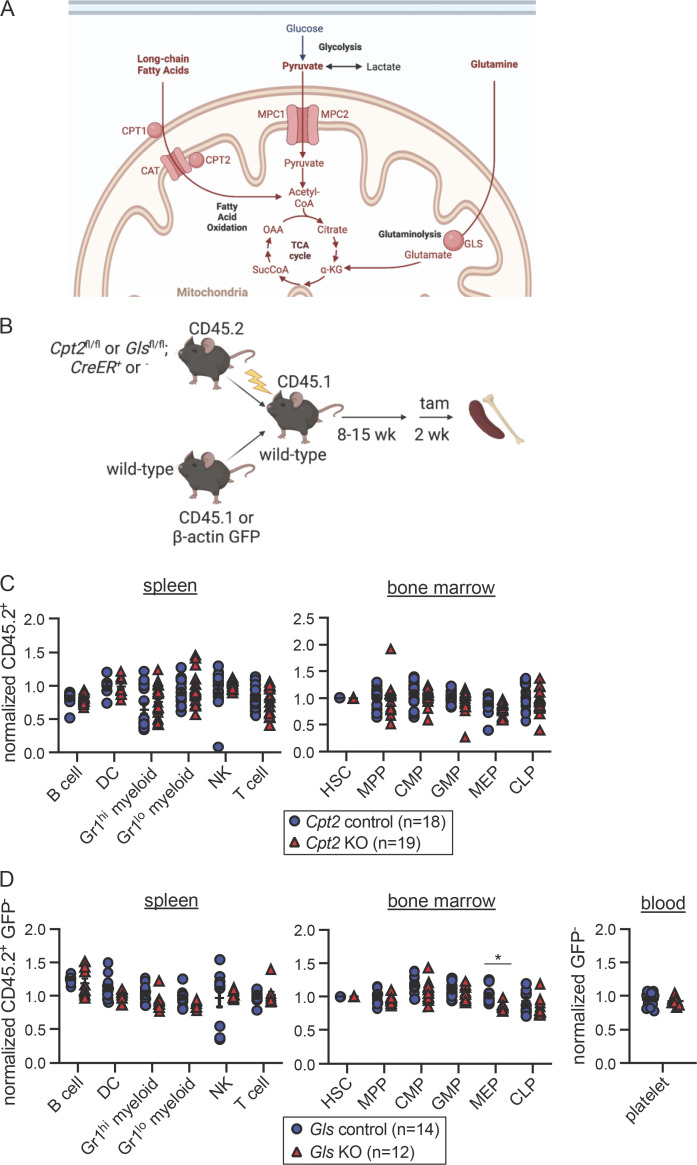
**Long-chain fatty acid oxidation and glutaminolysis are dispensable for most hematopoietic lineages. (A)** Schematic representation of relevant metabolic pathways. Pyruvate enters the mitochondria through the MPC, composed of subunits MPC1 and MPC2. Long-chain fatty acids enter the mitochondria via carnitine palmitoyltransferase 1 and 2 (CPT1 and CPT2) and carnitine acylcarnitine translocase (CAT). Within the mitochondria, GLS hydrolyzes glutamine into glutamate. **(B)** Schematic representation of mixed bone marrow chimera experiments to assess hematopoietic requirements for *Cpt2* control (*Cpt2*^*fl/fl*^*; ROSA26 CreER*^*−/−*^) or KO (*Cpt2*^*fl/fl*^*; ROSA26 CreER*^*+/+*^) cells and *Gls* control (*Gls*^*fl/fl*^*; ROSA26 CreER*^*−/−*^) or KO (*Gls*^*fl/fl*^*; ROSA26 CreER*^*+/+*^) cells. **(C)** CD45.2 chimerism was normalized to either pre-tamoxifen peripheral blood chimerism levels for the spleen or HSC chimerism for the bone marrow of *Cpt2* chimeras. Data are shown for B cells, DCs, Gr1^hi^ and Gr1^lo^ myeloid cells, NK cells, and T cells for the spleen. Data are shown for HSCs, MPPs, CMPs, GMPs, MEPs, and CLPs in the bone marrow. Each symbol represents an individual mouse. Mean values ± SEM are shown, and data are pooled from three independent experiments. P values >0.05 by two-way ANOVA with post-hoc Tukey’s multiple comparisons test are not depicted. **(D)** CD45.2^+^ GFP^−^ chimerism was normalized as stated in Fig. 1 C for *Gls* chimeras. Cell populations assessed are the same as in Fig. 1 C with the addition of platelets in the blood. Each symbol represents an individual mouse. Mean values ± SEM are shown, and data are pooled from two independent experiments. *, P < 0.05 by two-way ANOVA with post-hoc Tukey’s multiple comparisons test.

To define essential carbon sources that fuel the TCA cycle in hematopoietic lineages, we employed in vivo genetic ablation models. We focused on long-chain fatty acid oxidation, glutaminolysis, and mitochondrial pyruvate utilization as all these pathways are major contributors to ATP production in many other cell types. First, we used mice deficient in carnitine palmitoyltransferase 2 (CPT2), which transports long-chain fatty acids into the inner matrix of the mitochondria for oxidation (Fig. 1 A; Houten et al., 2016; Lee et al., 2015). Because germline deletion of *Cpt2* is lethal (Isackson et al., 2008; Ji et al., 2008; Longo et al., 2006; Nyman et al., 2005), we crossed *Cpt2*^*fl/fl*^ mice (Lee et al., 2015) to *ROSA26 CreER* animals, which constitutively express tamoxifen-inducible Cre recombinase (Ventura et al., 2007). WT CD45.1^+^ and *Cpt2*^*fl/fl*^*; ROSA26 CreER*^*−/−*^ or ^*+/+*^ CD45.2^+^ bone marrow cells were mixed in equal proportions and transplanted into irradiated recipients (Fig. 1 B). Reconstitution was allowed to proceed for at least 8 wk before the administration of tamoxifen to ablate *Cpt2* in CD45.2^+^ cells. This system avoids lethality following *Cpt2* deletion and allows assessment of blood lineage-intrinsic phenotypes following the restoration of mature cells after irradiation.

**Figure S1.**
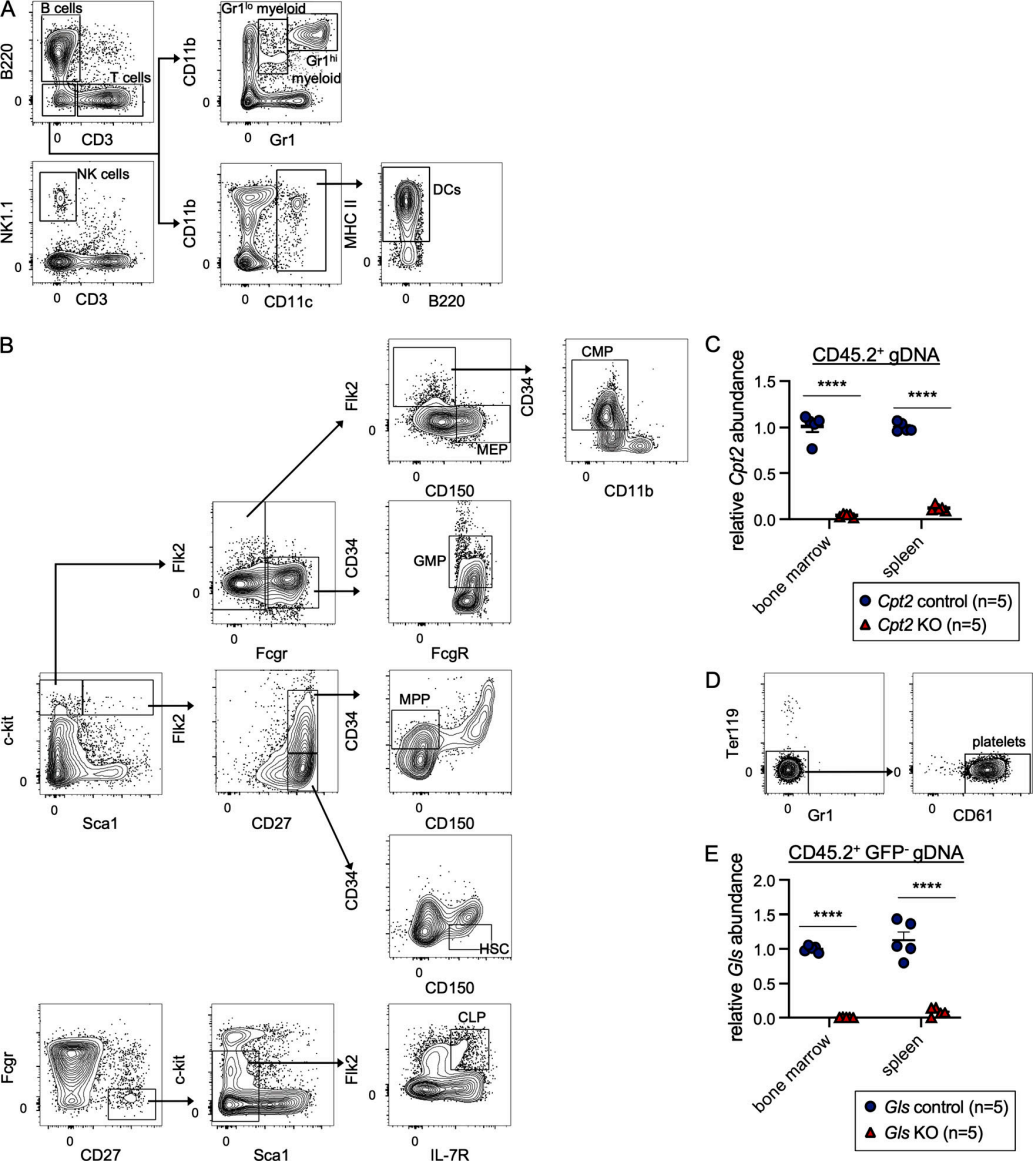
**Gating strategy and quantification of deletion of *Cpt2* and *Gls*. (A)** Representative flow cytometry gating strategy for splenic populations. **(B)** Representative flow cytometry gating strategy for bone marrow populations. **(C)** Quantitative PCR analysis of genomic *Cpt2* deletion. CD45.2^+^ cells from the bone marrow or spleen were sorted for genomic DNA extraction. DNA quantification within loxP sites was quantified relative to WT cells and GAPDH. Mean values ± SEM are shown. Data are pooled from two independent experiments. ****, P < 0.0001 by Student’s two-tailed *t* test. **(D)** Representative flow cytometry gating strategy for platelets in the blood. **(E)** Quantitative PCR analysis of genomic *Gls* deletion. CD45.2^+^ GFP^−^ cells from the bone marrow or spleen were sorted for genomic DNA extraction. DNA quantification within loxP sites was quantified relative to WT cells and GAPDH. Mean values ± SEM are shown. Data are pooled from two independent experiments. ****, P < 0.0001 by Student’s two-tailed *t* test.

**Figure S2.**
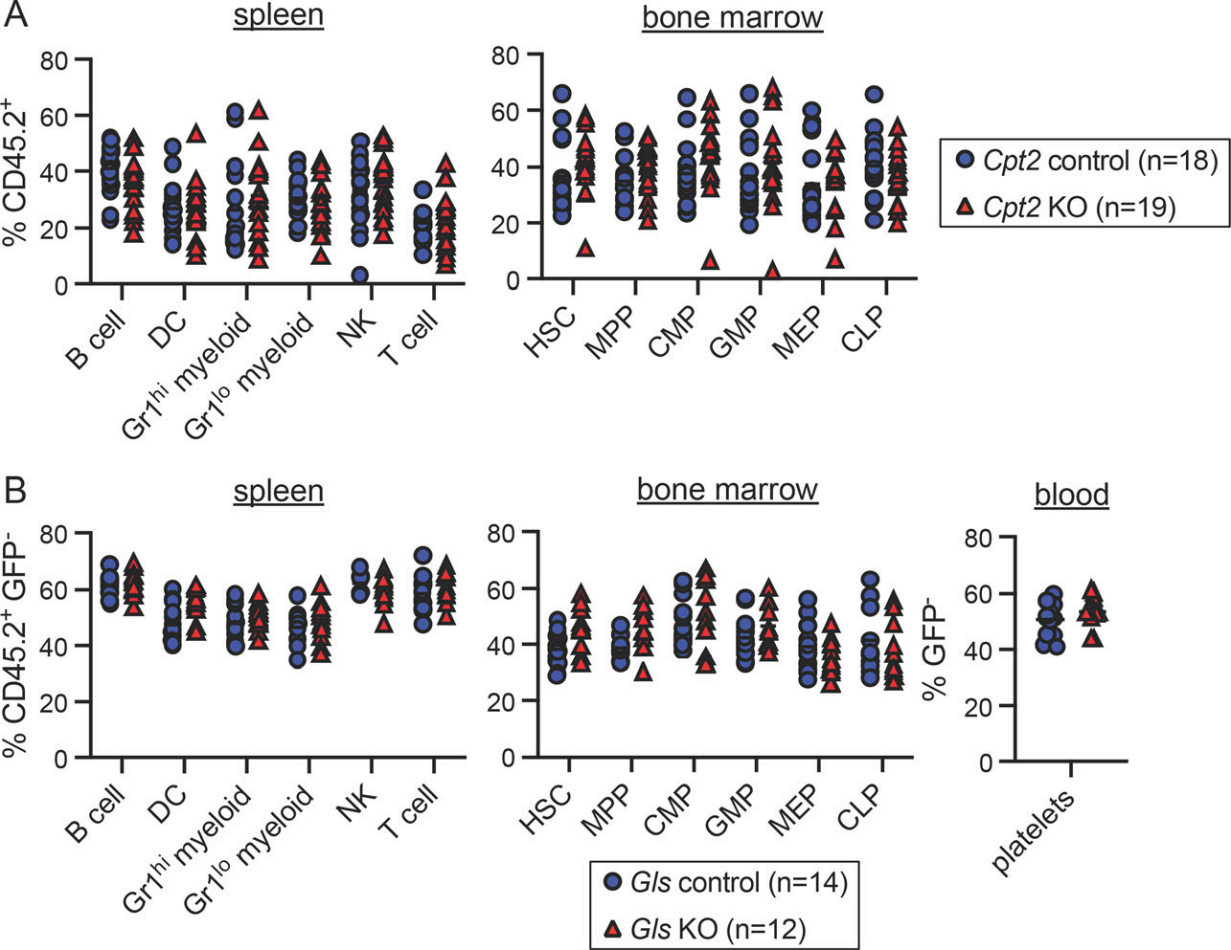
**Raw chimerism frequencies for *Cpt2* and *Gls* chimeras. (A)** Raw chimerism values for *Cpt2* chimeras shown in Fig. 1 C. Each symbol represents an individual mouse. Mean values ± SEM are shown, and data are pooled from three independent experiments. P values >0.05 by two-way ANOVA with post-hoc Tukey’s multiple comparisons test are not depicted. **(B)** Raw chimerism values for *Gls* chimeras shown in Fig. 1 D. Each symbol represents an individual mouse. Mean values ± SEM are shown, and data are pooled from two independent experiments. P values >0.05 by two-way ANOVA with post-hoc Tukey’s multiple comparisons test are not depicted.

After 2 wk of tamoxifen treatment, chimerism of hematopoietic populations in the spleen and bone marrow was assessed and normalized to the chimerism of either pre-tamoxifen blood lineages for splenic populations or HSCs for bone marrow progenitors. This normalization corrects for variability in chimerism between animals prior to tamoxifen-induced deletion, which typically ranged from 40 to 60%. Analysis of the spleen included B cells, dendritic cells (DCs), Gr1^hi^ myeloid cells, Gr1^lo^ myeloid cells, NK cells, and T cells (gating strategy in Fig. S1 A). Bone marrow populations included HSCs, Flk2^+^ multipotent progenitors (MPPs; Christensen and Weissman, 2001; Adolfsson et al., 2001), Flk2^+^ common myeloid progenitors (CMPs; Karsunky et al., 2003), granulocyte-macrophage progenitors (GMPs; Akashi et al., 2000; Becker et al., 2012), megakaryocyte-erythroid progenitors (MEPs; Pronk et al., 2007), and common lymphoid progenitors (CLPs; gating strategy in Fig. S1 B; Inlay et al., 2009). Despite efficient *Cpt2* deletion (Fig. S1 C), we found no differences between *Cpt2* KO cells and WT controls (Fig. 1 C; raw chimerism values in Fig. S2 A). This suggests that long-chain fatty acid oxidation is not required for the development or maintenance of lymphoid or myeloid cells and that other pathways such as glutaminolysis or pyruvate metabolism may be more important for these lineages. Prior studies have suggested an important role for glutaminolysis in human erythropoiesis (Oburoglu et al., 2014), but other lineages have not yet been examined.

Glutaminase (GLS) is an aminohydrolase that catalyzes the conversion of glutamine to glutamate, which can then be converted into the TCA cycle intermediate α-ketoglutarate (Fig. 1 A; Curthoys and Watford, 1995). A potential compensatory enzyme, GLS2, is minimally expressed within the hematopoietic system (Heng et al., 2008). Since germline *Gls* deletion causes lethality (Masson et al., 2006), we generated competitive bone marrow chimeras using *Gls*^*fl/fl*^ mice (Mingote et al., 2016). Given that erythrocytes, megakaryocytes, and platelets lack CD45 expression, we generated mixed chimeras for *Gls* using β-actin GFP transgenic mice as WT competitors (Fig. 1 B). GFP expression, though minimal in mature erythrocytes, can be detected in MEPs and in mature platelets (Forsberg et al., 2006; Wright et al., 2001). At the end of tamoxifen treatment, the same cell subsets examined in *Cpt2*-deleted chimeras were analyzed alongside platelets in the blood (gating strategy in Fig. S1, A–D). Deletion of *Gls* was efficient (Fig. S1 E) but did not affect lymphoid or myeloid cell chimerism (Fig. 1 D; raw chimerism values in Fig. S2 B). GLS-deficient MEP chimerism was slightly reduced (about 10%, P < 0.05) but platelets were unaffected (Fig. 1 D and Fig. S2 B). Together these data demonstrate that long-chain fatty acid oxidation and glutaminolysis are largely dispensable for lymphoid and myeloid cells, while erythroid progenitors have a partial dependence on glutaminolysis. These data suggest that other carbon sources, such as pyruvate, may be more important for hematopoiesis.

### Mitochondrial pyruvate is transiently required by both myeloid progenitors and mature cells

MPC1 and MPC2 are subunits of the heteromeric mitochondrial pyruvate carrier (MPC), both of which are necessary for the transport of pyruvate into the mitochondria (Fig. 1 A; Bricker et al., 2012; Herzig et al., 2012). As with *Cpt2* and *Gls*, germline deletion of either subunit of MPC is lethal (Vigueira et al., 2014). During the course of prior work on the metabolism of antibody-secreting cells (Lam et al., 2016), we preliminarily observed that neutrophils were reduced upon *Mpc2* ablation in mixed chimeras. Other lymphoid lineages were not obviously impacted, at least at short timepoints after *Mpc2* deletion. To investigate further, we generated mixed bone marrow chimeras of WT CD45.1^+^ and CD45.2^+^ *Mpc2*^*fl/fl*^*; ROSA26 CreER*^*−/−*^ or *CreER*^*+/−*^ genotypes (McCommis et al., 2015). A more comprehensive analysis was then performed compared with our earlier results to quantify other lineages, progenitors, and the kinetics of blood lineage chimerism changes following *Mpc2* ablation.

**Figure 2.**
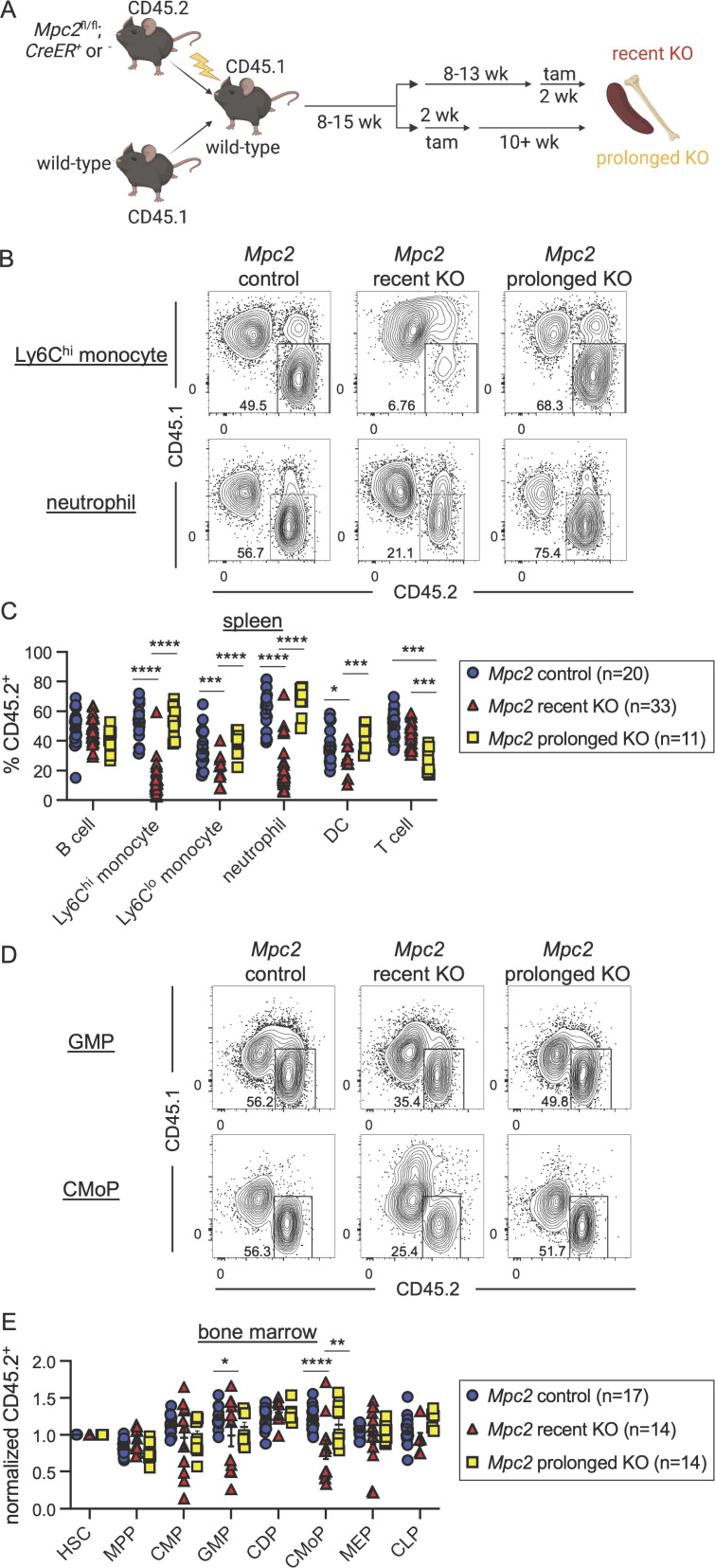
**Mitochondrial pyruvate is transiently required by both myeloid progenitors and mature cells. (A)** Schematic representation of mixed bone marrow chimeras to assess the hematopoietic requirement for *Mpc2* (*Mpc2*^*fl/fl*^*; ROSA26 CreER*^*−/−*^ or *ROSA26 CreER*^*+/−*^), both immediately following deletion and after prolonged deletion (10+ wk after tamoxifen). **(B)** Representative flow cytometry plots of CD45.2 chimerism of Ly6C^hi^ monocytes and neutrophils for *Mpc2* control, recent KO, and prolonged KO chimeras. **(C)** CD45.2 chimerism of splenic immune lineages at early or prolonged timepoints after deletion of *Mpc2*. Each symbol represents an individual mouse. Mean values ± SEM are shown. Data are pooled from four independent experiments. *, P < 0.05; ***, P < 0.001; and ****, P < 0.0001 by two-way ANOVA with post-hoc Tukey’s multiple comparisons test. **(D)** Representative flow cytometry plots of CD45.2 chimerism of GMPs and CMoPs. **(E)** CD45.2 chimerism of bone marrow progenitors described previously with the addition of CMoPs and CDPs at early or prolonged timepoints after deletion of *Mpc2.* Data are normalized to HSC CD45.2 chimerism. Each symbol represents an individual mouse. Mean values ± SEM are shown, and data are pooled from three independent experiments. *, P < 0.05; **, P < 0.01; ****, P < 0.0001 by two-way ANOVA with post-hoc Tukey’s multiple comparisons test.

**Figure S3.**
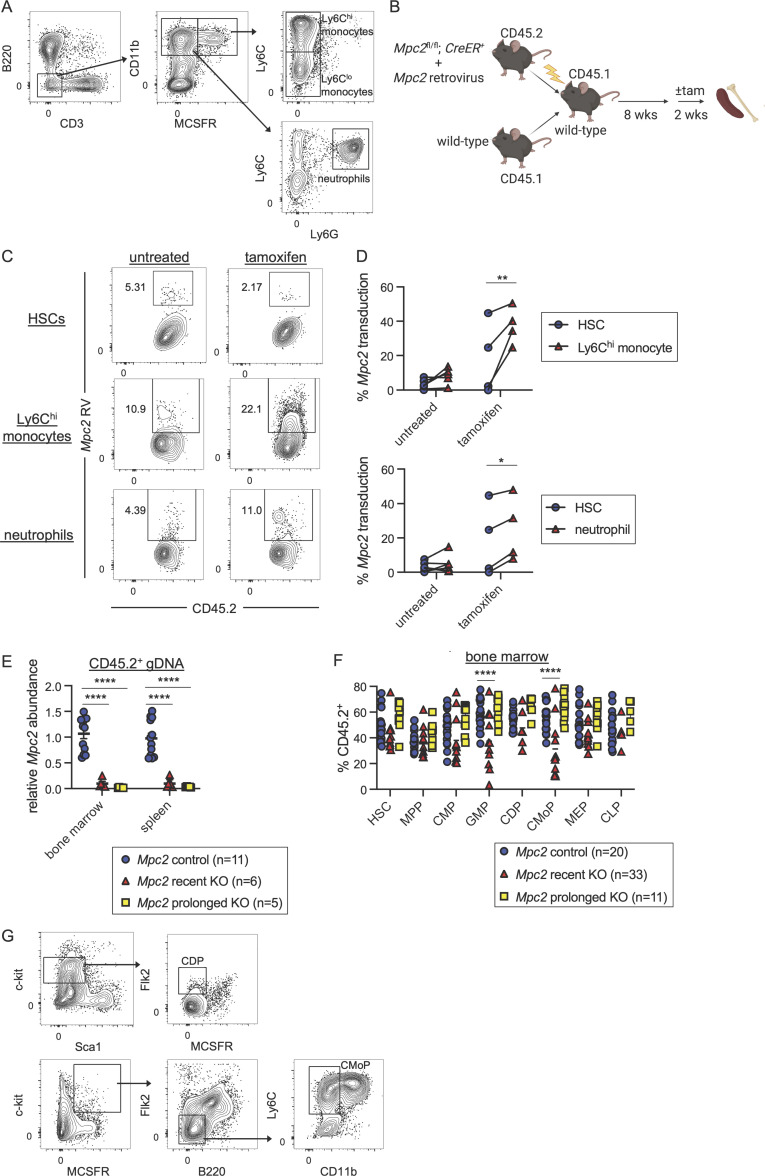
**Gating strategy, quantification of deletion of *Mpc2*, and *Mpc2* retroviral rescue of MPC2-deficient myeloid cells. (A)** Representative flow cytometry gating strategies for splenic Ly6C^hi^ and Ly6C^lo^ monocytes and neutrophils. **(B)** Schematic representation of *Mpc2* retroviral bone marrow chimera experiments to assess ability to rescue *Mpc2* KO (+tam) or control (no tam) Ly6C^hi^ monocytes and neutrophils. C-kit^+^ cells were enriched from the bone marrow of *Mpc2*^*fl/fl*^*; ROSA26 CreER*^*+/−*^ mice and transduced with *Mpc2* retrovirus prior to transplantation alongside bone marrow from WT mice into irradiated recipients. **(C)** Representative flow cytometry plots of *Mpc2* retrovirus (RV) expression in HSCs, Ly6C^hi^ monocytes, and neutrophils of an individual control or KO mouse. **(D)** *Mpc2* transduction in HSCs paired with Ly6C^hi^ monocytes (top) or neutrophils (bottom) from the same mouse (*n* = 6 for untreated and *n* = 4 for tamoxifen). Data are pooled from two independent experiments. *, P < 0.05; **, P < 0.01 by Student’s two-tailed paired *t* test. **(E)** Quantitative PCR analysis of genomic *Mpc2* deletion in *Mpc2* chimeras. CD45.2^+^ cells from the bone marrow or spleen were sorted for genomic DNA extraction. DNA quantification within loxP sites was quantified relative to WT cells and GAPDH. Mean values ± SEM are shown. Data are pooled from two independent experiments. ****, P < 0.0001 by one-way ANOVA with post-hoc Tukey’s multiple comparisons test. **(F)** Raw chimerism values for *Mpc2* chimeras shown in Fig. 2 E. Each symbol represents an individual mouse. Mean values ± SEM are shown, and data are pooled from three independent experiments. ****, P < 0.0001 by two-way ANOVA with post-hoc Tukey’s multiple comparisons test. **(G)** Representative flow cytometry gating strategies for CDPs and CMoPs in the bone marrow.

After reconstitution, half of the chimeras were given tamoxifen and then sacrificed at least 10 wk later (prolonged KO; Fig. 2 A). This group allows for the assessment of longer-term effects on lymphoid lineages, which turn over infrequently. The other half was given tamoxifen 2 wk prior to euthanasia (recent KO). Consistent with our prior observations, the recent KO group was significantly reduced in neutrophil chimerism (Fig. 2, B and C; gating strategy in Fig. S3 A). Ly6C^hi^ and Ly6C^lo^ monocytes were also substantially reduced immediately following *Mpc2* deletion, while DCs were slightly reduced (Fig. 2, B and C). *Mpc2*-deficient neutrophils and monocytes were rescued by *Mpc2*-encoding retrovirus (Fig. S3, B–D), demonstrating that the phenotype was not due to Cre or tamoxifen toxicity. B cell chimerism was not affected in either group, whereas *Mpc2*-deficient T cell chimerism was diminished only in the prolonged KO group (Fig. 2 C). Yet contrary to our expectations, after prolonged deletion of *Mpc2*, neutrophils, Ly6C^hi^ monocytes, and Lyc6^lo^ monocytes all recovered to mirror WT controls (Fig. 2 C). We considered the possibility that the myeloid recovery was driven by the preferential expansion of cells that had incompletely deleted *Mpc2.* However, this seems unlikely given that this recovery would occur in competition against CD45.1^+^ WT cells. Indeed, *Mpc2* remained fully deleted following prolonged deletion in both the spleen and bone marrow (Fig. S3 E).

In the recent KO group, there was a modest reduction in *Mpc2*-deficient GMPs and a more substantial reduction in common monocyte progenitors (CMoPs; Fig. 2, D and E; raw chimerism values in Fig. S3 F; gating strategy in Fig. S3 G; Hettinger et al., 2013). MPP and CMP chimerism remained unaffected, as did that of common DC progenitors (CDPs; Naik et al., 2007; Onai et al., 2007), MEPs, and CLPs (Fig. 2 E and Fig. S3 F). Consistent with what was observed in mature myeloid cells, progenitor chimerism was unaffected following prolonged *Mpc2* deletion (Fig. 2 E and Fig. S3 F). These results suggest that MPC2 is required for the homeostasis of the myeloid lineage, but that these cells can somehow adapt over time to the loss of mitochondrial pyruvate metabolism.

### Mature myeloid cells diminish then rapidly recover after *Mpc2* deletion

**Figure 3.**
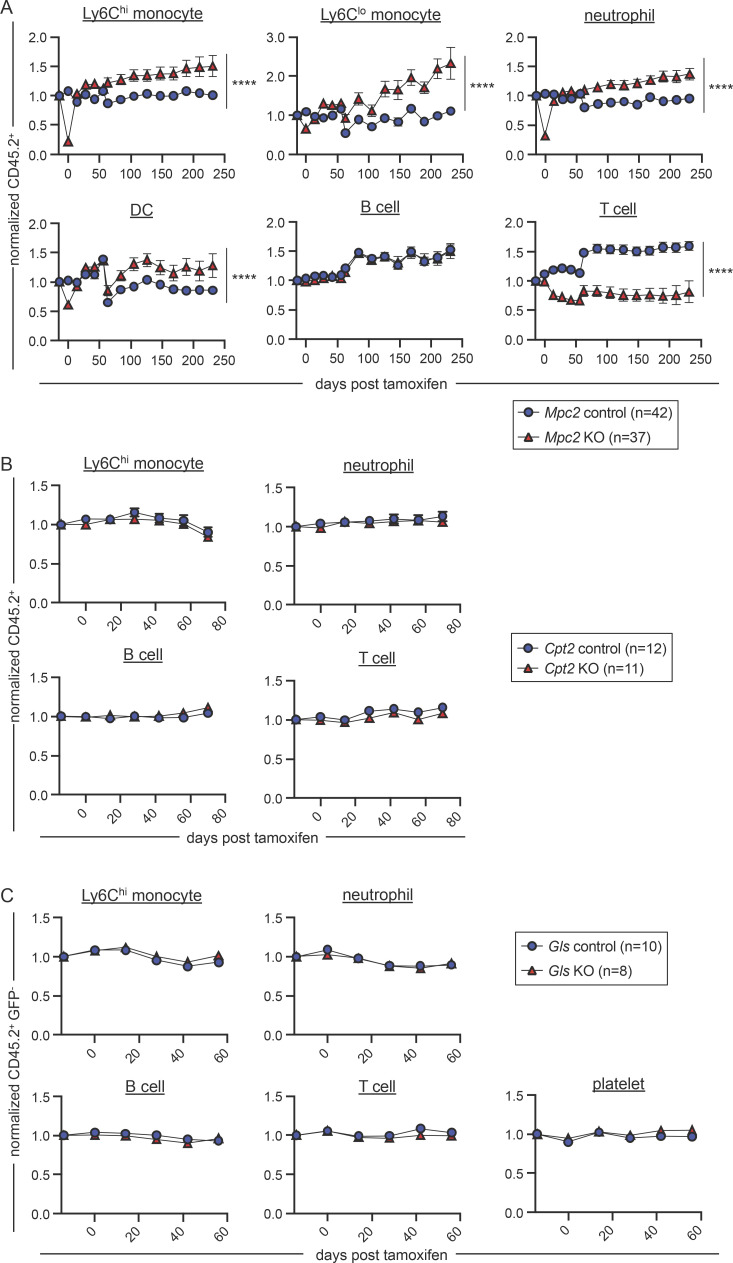
**Mature myeloid cells diminish then rapidly recover after *Mpc2* deletion. (A)** CD45.2 peripheral blood chimerism of mature cell populations was assessed every 2–3 wk in *Mpc2* chimeras. Values are normalized to pre-tamoxifen chimerism of each cell type. Data are pooled from three independent experiments. Mean values ± SEM are shown. ****, P < 0.0001 by paired two-way ANOVA with post-hoc Tukey’s multiple comparisons test. **(B and C)** CD45.2 peripheral blood chimerism of mature cell populations was assessed every 2 wk in *Cpt2* (B) or *Gls* (C) chimeras. Values are normalized to pre-tamoxifen chimerism of each cell type. Mean values ± SEM are shown. Data are pooled from two independent experiments. P values >0.05 by two-way ANOVA with post-hoc Tukey’s multiple comparisons test are not depicted.

**Figure S4.**
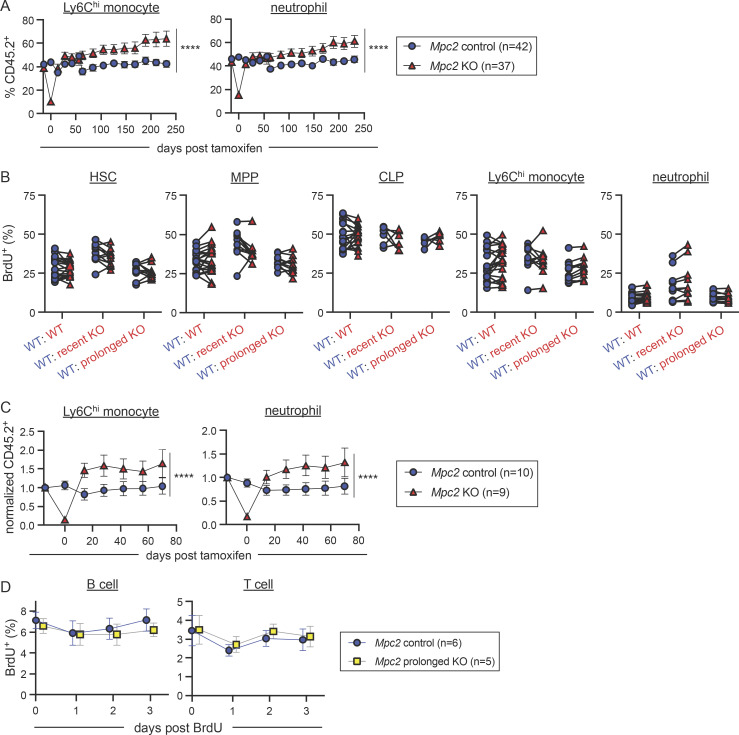
**Proliferation, survival, and recovery of MPC2-deficient cells. (A)** Raw chimerism values for peripheral myeloid cells of *Mpc2* chimeras in Fig. 3 A. Data are pooled from three independent experiments. Mean values ± SEM are shown. ****, P < 0.0001 by paired two-way ANOVA with post-hoc Tukey’s multiple comparisons test. **(B)** BrdU incorporation was measured in both CD45.1^+^ (WT) and CD45.2^+^ (WT, recent *Mpc2* deletion, or prolonged *Mpc2* deletion) HSCs, MPPs, CLPs, Ly6C^hi^ monocytes, and neutrophils following a 1-h pulse of BrdU. Each line connects CD45.1^+^ and CD45.2^+^ cells within the same mouse (*n* = 11 for WT chimeras and *n* = 19 for recent and prolonged KO chimeras). Data are pooled from two independent experiments. P values >0.05 by paired two-way ANOVA with post-hoc Sidak’s multiple comparisons test are not depicted. **(C)** *Mpc2* chimeras were setup at a ratio of 90% WT to 10% *Mpc2*^*fl/fl*^*; ROSA26 CreER*^*−/−*^ or *CreER*^*+/−*^ bone marrow cells (as opposed to the 1:1 ratio in Fig. 3). CD45.2 peripheral blood chimerism of Ly6C^hi^ monocytes and neutrophils was assessed every 2 wk in *Mpc2* chimeras. Values are normalized to pre-tamoxifen chimerism of each cell type. Mean values ± SEM are shown. ****, P < 0.0001 by paired two-way ANOVA with post-hoc Tukey’s multiple comparisons test. **(D)** BrdU incorporation was measured in peripheral CD45.2^+^ B and T cells for 3 d following a week of BrdU water administration. Mean values ± SEM are shown. Data are pooled from two independent experiments. P values >0.05 by two-way ANOVA with post-hoc Tukey’s multiple comparisons test are not depicted.

Given that myeloid cells are initially depleted upon *Mpc2* deletion but recover by 10 wk, we performed experiments to define the kinetics of this recovery. Following over 8 wk of reconstitution, *Mpc2* chimeras were administered tamoxifen and then were bled every 2–3 wk to monitor donor contribution over time relative to their pre-tamoxifen chimerism. Rather than a gradual recovery, we observed a sharp increase in *Mpc2*-deficient myeloid cells within 2 wk after the initial nadir (Fig. 3 A; raw chimerism values in Fig. S4 A). These cells then further expanded beyond their pre-tamoxifen levels. In contrast, the reduction in *Mpc2*-deficient T cell chimerism was observed at 2 wk after tamoxifen and remained relatively stable throughout the duration of the experiment (Fig. 3 A). This is consistent with the requirement of MPC1 for αβ T cell development in the thymus (Ramstead et al., 2020). Similarly, pyruvate dehydrogenase is also required for thymic T cell development (Jun et al., 2021). B cells were not affected by the loss of *Mpc2* at any timepoint (Fig. 3 A). We additionally examined *Cpt2* and *Gls* chimeras over time to determine if a defect would become apparent later after deletion. Yet no defects were observed for at least 60–80 d (Fig. 3, B and C). These data suggest that myeloid cells are dependent on mitochondrial pyruvate import yet can adjust to the absence of MPC2 over time.

### GMPs and CMoPs proliferate rapidly immediately following *Mpc2* deletion

**Figure 4.**
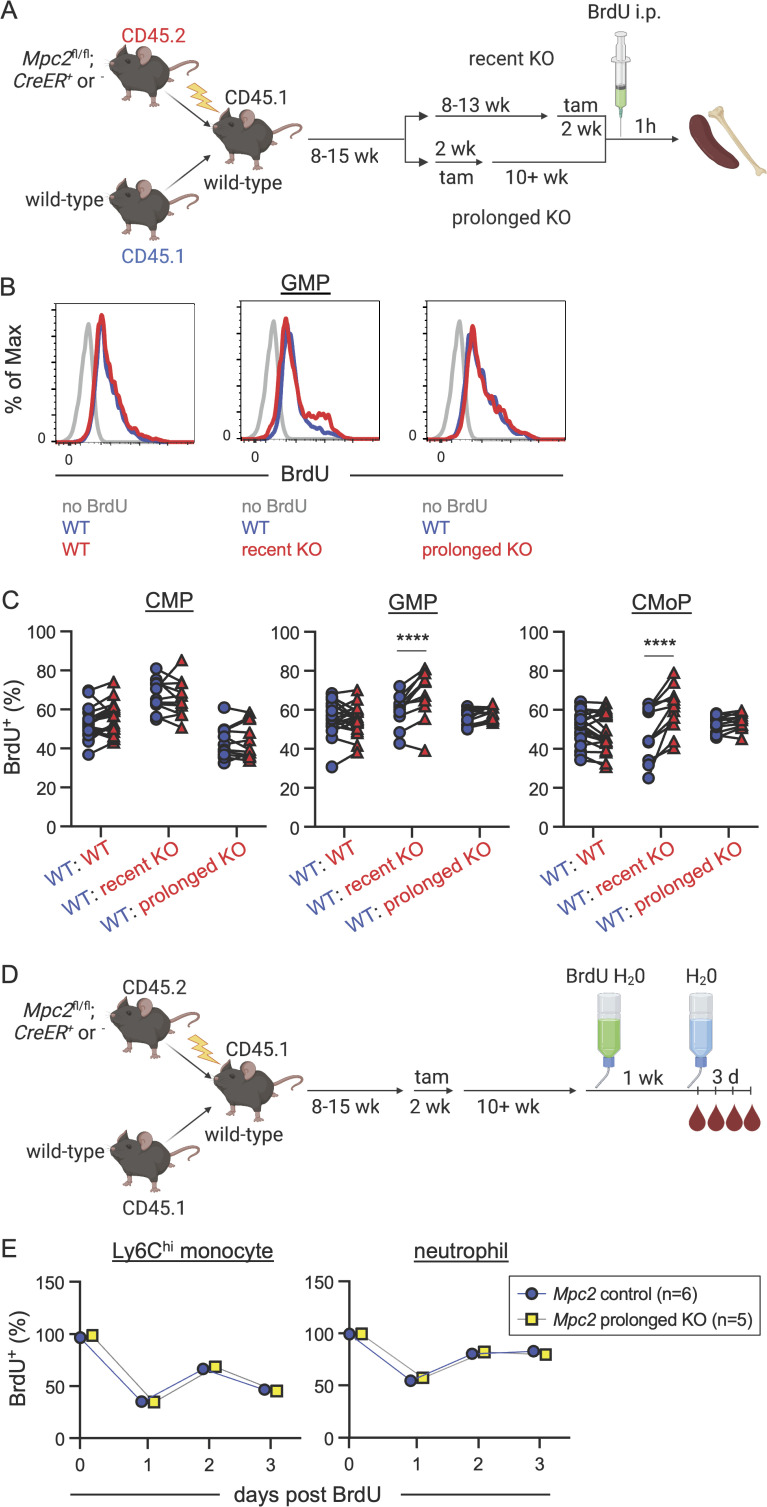
**GMPs and CMoPs proliferate rapidly immediately following *Mpc2* deletion. (A)** Schematic representation of BrdU pulse experiment using *Mpc2* mixed bone marrow chimeras. Chimeric mice were injected with BrdU 1 h prior to sacrifice. **(B)** Representative histograms of BrdU staining in GMPs of *Mpc2* control, recent KO, and prolonged KO chimeras. Gray lines represent staining of GMPs from a mouse that was not injected with BrdU. Blue and red lines represent CD45.1^+^ (WT) and CD45.2^+^ (WT, recent KO, or prolonged KO) GMPs respectively from the same mouse injected with BrdU. **(C)** BrdU incorporation was measured in both CD45.1^+^ (WT) and CD45.2^+^ (WT, recent *Mpc2* deletion, or prolonged *Mpc2* deletion) CMPs, GMPs, and CMoPs following a 1-h pulse of BrdU. Each line connects CD45.1^+^ and CD45.2^+^ cells within the same mouse (*n* = 11 for WT chimeras and *n* = 19 for recent and prolonged KO chimeras). Data are pooled from two independent experiments. ****, P < 0.0001 by paired two-way ANOVA with post-hoc Sidak’s multiple comparisons test. **(D)** Schematic representation of BrdU pulse-chase experiment using *Mpc2* mixed bone marrow chimeras to assess survival and turnover. Chimeras were administered BrdU water for 1 wk and then were switched back to normal drinking water, at which point BrdU incorporation was assessed in peripheral blood at the time of removal from BrdU and three successive days afterwards. **(E)** BrdU incorporation was measured in peripheral CD45.2^+^ Ly6C^hi^ monocytes and neutrophils following a 3-d chase after a week of BrdU water administration. Mean values ± SEM are shown. Data are pooled from two independent experiments. P values >0.05 by two-way ANOVA with post-hoc Tukey’s multiple comparisons test are not depicted.

Given the recovery we observed in myeloid cells following the deletion of *Mpc2*, we next performed experiments to determine if MPC2-deficient cells were proliferating more and/or surviving longer to facilitate this recovery. To determine if the proliferation of myeloid progenitors was altered by the loss of *Mpc2*, we injected chimeras with BrdU 1 h prior to sacrifice (Fig. 4 A). We then compared BrdU incorporation in the WT CD45.1^+^ cells with control, recent KO, or prolonged KO CD45.2^+^ cells within the same animals. Recent MPC2-deficient GMPs and CMoPs proliferated significantly more than did WT GMPs and CMoPs within the same mouse, while proliferation was similar between MPC2-deficient and -sufficient CMPs (Fig. 4, B and C). After prolonged deletion of *Mpc2*, proliferation of these populations was similar to that of their WT counterparts (Fig. 4, B and C). No changes were observed in the proliferation of MPC2-deficient HSCs, MPPs, CLPs, or mature myeloid cells (Fig. S4 B). These data demonstrate that MPC2 deficiency triggers an adaptation in GMPs and CMoPs that is accompanied by a transient burst in proliferation. This allows for rapid regeneration of themselves and mature myeloid populations. After prolonged *Mpc2* deletion, this proliferative burst subsides.

Prior studies have shown that upstream hematopoietic progenitors can sense and respond to the loss of downstream mature lineages by increasing proliferation (Boettcher and Manz, 2017; Cheshier et al., 2007; Takizawa et al., 2012). We considered the possibility that MPC2-deficient progenitors are better at sensing and proliferating in response to mature cell loss than their WT counterparts. Alternatively, the initial proliferative burst in MPC2-deficient progenitors may occur cell-intrinsically, perhaps due to a metabolic switch. To distinguish between these alternatives, we generated mixed bone marrow chimeras at a 90:10 ratio of WT to *Mpc2* KO cells. In this setting, the absolute loss in mature myeloid cells is minimal and would not be expected to trigger feedback to upstream progenitors. We observed a similar reduction in mature myeloid cells immediately following *Mpc2* deletion with a full recovery 2 wk later (Fig. S4 C. This suggests that the recovery is not due to a heightened sensing of mature cell loss but rather proliferation triggered by a cell-intrinsic metabolic switch.

We next performed a BrdU pulse-chase experiment to determine if the recovery of MPC2-deficient cells is also fueled by enhanced survival. Due to the infrequency of mature myeloid cells following the recent deletion of *Mpc2*, we focused on survival in the prolonged KO group. Chimeras were administered BrdU in their drinking water for 1 wk (Fig. 4 D). At the end of the week, mice were switched back to normal drinking water and were bled to establish baseline BrdU incorporation. The mice were then bled for three consecutive days following the cessation of BrdU administration to determine cell survival time and turnover. There were no differences observed in BrdU retention over time in peripheral Ly6C^hi^ monocytes or neutrophils between control mice or MPC2-deficient mice after prolonged deletion (Fig. 4 E). As anticipated, B and T cell survival was also unaffected by *Mpc2* deletion (Fig. S4 D). Taken together, these data suggest that the MPC2-deficient myeloid recovery is due specifically to increased myeloid progenitor proliferation fueled by an intrinsic metabolic switch and is not due to enhanced mature cell survival.

### GMPs use glutamine in vitro to generate TCA cycle intermediates

**Figure 5.**
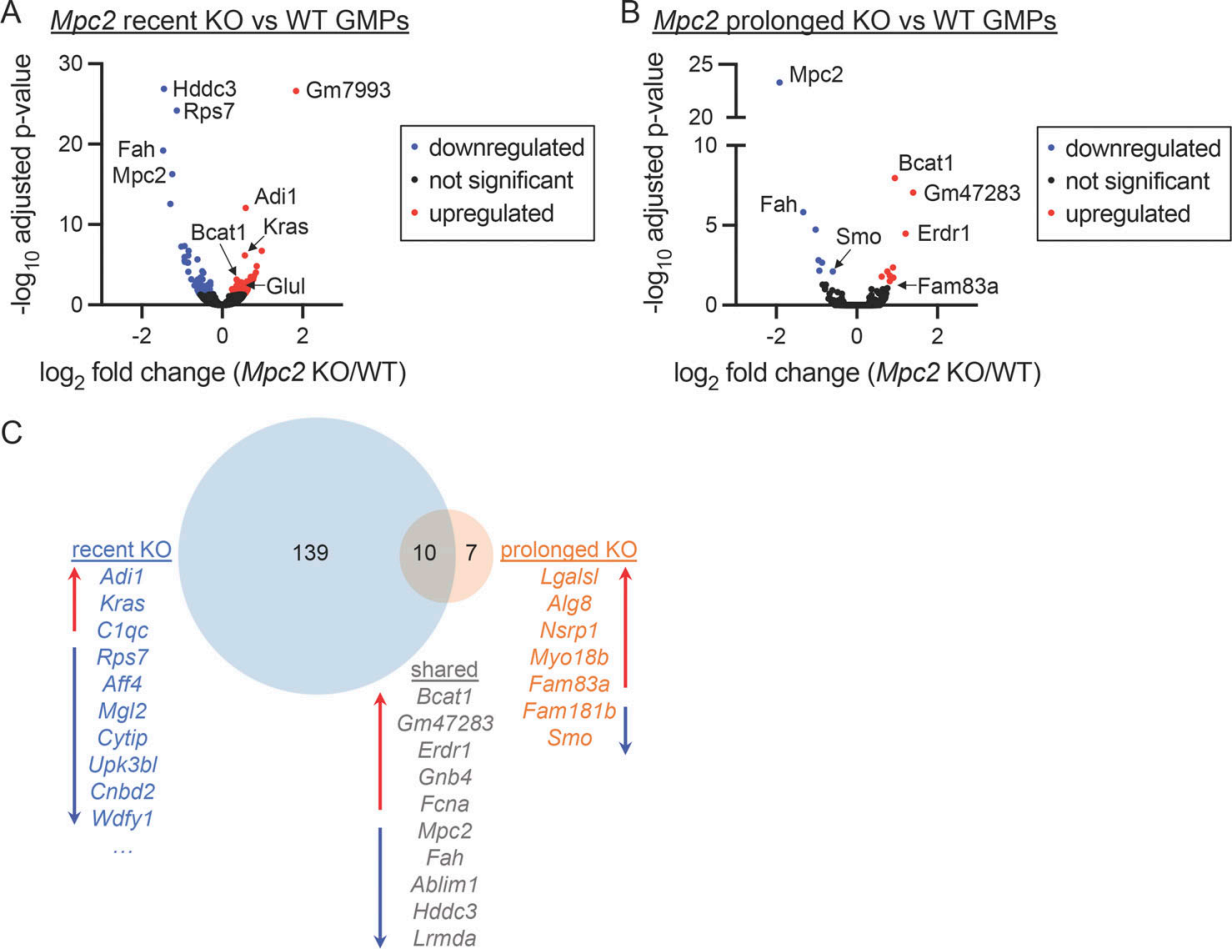
**GMPs differentially express numerous genes immediately following *Mpc2* deletion but few persist following myeloid recovery. (A and B)** Volcano plots of gene expression fold changes between *Mpc2* recent KO and WT GMPs (A) or *Mpc2* prolonged KO and WT GMPs (B). Following RNA-seq, adjusted P values were calculated using DESeq2. Each dot represents a gene. Recent *Mpc2* KO mice were pooled into groups of two mice to obtain a sufficient number of cells. A total of 10 mice were used but were processed as five samples. For all other groups, five mice were analyzed without pooling for each genotype. **(C)** Venn diagram analysis depicting genes differentially expressed by only recent *Mpc2* KO GMPs, prolonged *Mpc2* KO GMPs, or both relative to WT GMPs. Lists of genes include all hits or the top 10 hits not including pseudogenes or predicted genes. Red up arrows indicate genes upregulated in KOs relative to WT, while blue down arrows indicate genes downregulated in KOs relative to WT. All data are available in the NCBI GEO (recent KO accession number GSE225578 and prolonged KO accession number GSE184548).

The rapid proliferation of GMPs and CMoPs immediately following the loss of the MPC suggests that these progenitors switch to another carbon source to survive and expand to meet the sudden demand to produce more mature myeloid cells. Such metabolic switches have been observed in other cell types and model organisms (Bensard et al., 2020; Schell et al., 2014; Vanderperre et al., 2016; Yang et al., 2014). To begin to define an alternate carbon source for MPC2-deficient myeloid progenitors and to determine whether the switch is driven by a transcriptional adaptation, we performed RNA sequencing (RNA-seq) on GMPs from control or *Mpc2* KO chimeras. Immediately following the deletion of *Mpc2* in GMPs, the expression of 149 genes was significantly different relative to WT GMPs (Fig. 5 A). Notably, *Glul*, which converts glutamate and ammonia into glutamine (Deuel et al., 1978; Tate and Meister, 1971), was upregulated ∼1.5-fold in MPC2-deficient GMPs relative to control GMPs (Fig. 5 A). *Bcat1*, which transfers α-amino groups from branched-chain amino acids (BCAAs; leucine, isoleucine, and valine) to α-ketoglutarate to generate α-ketoacids and glutamate (Hutson et al., 1988; Ichihara and Koyama, 1966), was also modestly upregulated in MPC2-deficient GMPs (Fig. 5 A). Yet only 17 genes, including *Mpc2*, were significantly altered in GMPs following prolonged deletion of *Mpc2* (Fig. 5 B). Of the differentially expressed genes, 10, including *Bcat1*, were shared between both recent and prolonged deletion groups (Fig. 5 C). KEGG pathway analyses were performed for both RNA-seq datasets but no pathways were identified with q < 0.05 and more than five gene hits.

**Figure 6.**
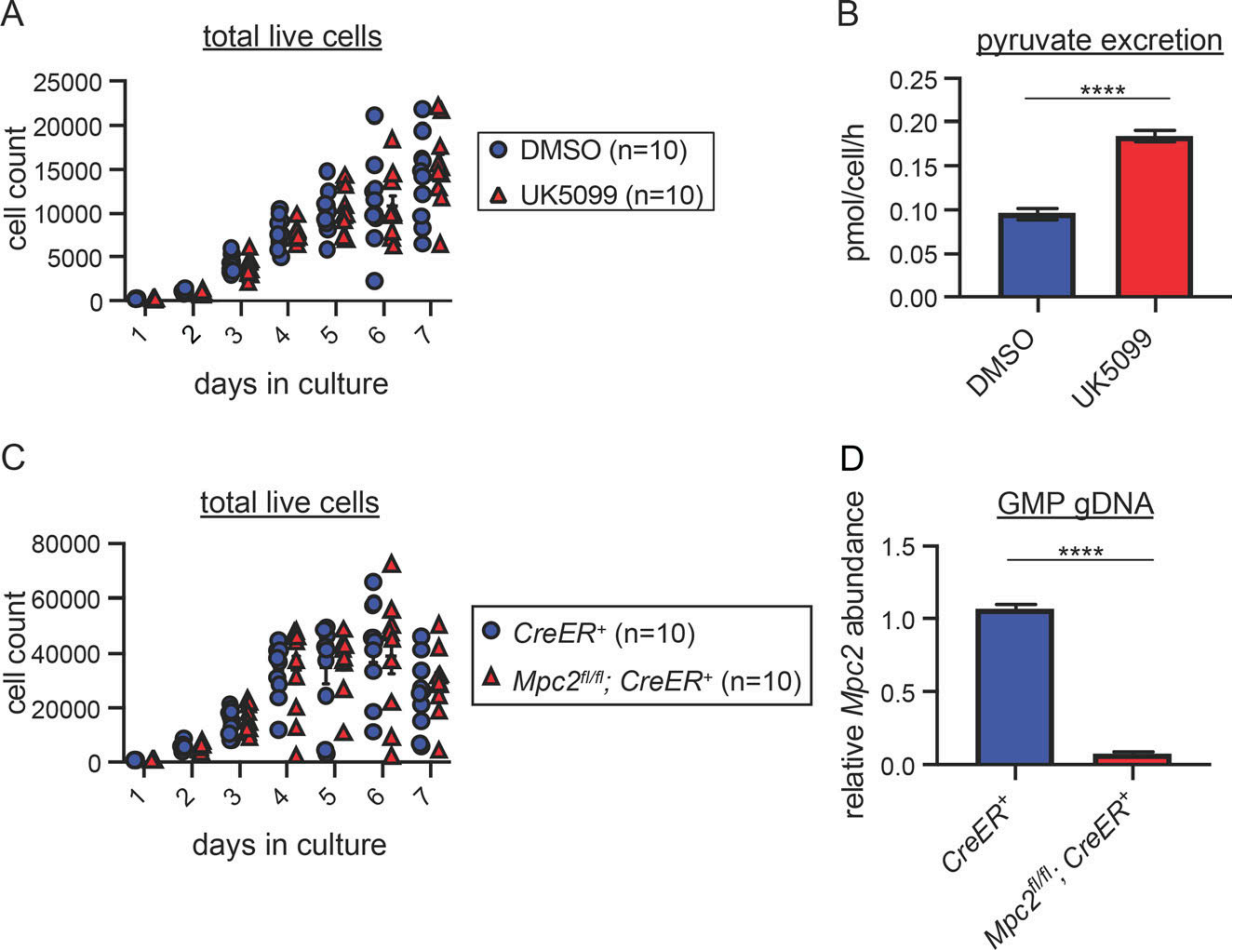
**In vitro GMPs do not require the MPC for survival. (A)** WT GMPs were cultured with DMSO or 10 μM UK5099 for 7 d. On each day of the culture, cells were harvested, and live cells were counted. One experiment with 10 technical replicates from two mice per group is depicted as a representative of four independent experiments. P values >0.05 by two-way ANOVA with post-hoc Tukey’s multiple comparisons test are not depicted. **(B)** Pyruvate excretion rate in the media of GMPs cultured with DMSO or UK5099 over a 48-h culture. ****, P < 0.0001 by Student’s two-tailed *t* test. Mean values ± SEM are shown for four replicates from two independent experiments. **(C)** GMPs from *Mpc2*^*fl/fl*^*; ROSA26 CreER*^*+/−*^ or *ROSA26 CreER*^*+/−*^ mice were treated with 0.5 μM 4-OHT. On each day of the culture, cells were harvested, and live cells were counted. One experiment with 10 technical replicates from two mice per group is depicted as a representative of two independent experiments. P values >0.05 by Student’s two-tailed *t* test are not depicted. **(D)** Quantitative PCR analysis of genomic *Mpc2* deletion in GMPs from *Mpc2*^*fl/fl*^*; ROSA26 CreER*^*+/−*^ or *ROSA26 CreER*^*+/−*^ mice. GMPs were treated with 4-OHT for 2 d prior to DNA extraction. DNA quantification within loxP sites was quantified relative to WT cells and GAPDH. Mean values ± SEM are shown for four replicates from two independent experiments. ****, P < 0.0001 by one-way ANOVA with post-hoc Tukey’s multiple comparisons test.

Given the transcriptional upregulation of *Bcat1* and *Glul* in MPC2-deficient GMPs, we employed an in vitro differentiation assay to determine whether these progenitors switch to oxidation of glutamine, BCAAs, or other carbon sources. GMPs were sorted from WT mice and cultured with stem cell factor (SCF) and GM-CSF to induce proliferation and granulocyte and myeloid differentiation. In contrast to our in vivo data, which show a clear requirement for mitochondrial pyruvate, treatment of cultures with the MPC inhibitor UK5099 (Halestrap and Denton, 1975) had no impact on cell survival over a 7-d period (Fig. 6 A). Treatment with UK5099 did increase the amount of pyruvate excreted in the cell culture media (Fig. 6 B), demonstrating successful inhibition of MPC. For genetic confirmation of these results, we sorted GMPs from *Mpc2*^*fl/fl*^*; CreER*^*+*^ and *CreER*^*+*^ mice and cultured them with 4-hydroxytamoxifen (4-OHT) to induce *Mpc2* deletion in floxed cells. No differences in cell counts were observed between MPC2-deficient and control cells despite efficient deletion of *Mpc2* (Fig. 6, C and D). These results are consistent with work showing marked differences between in vivo and in vitro metabolism in other systems (Cheng et al., 2011; Davidson et al., 2016; DeBerardinis et al., 2007; Faubert and DeBerardinis, 2017; Sellers et al., 2015). Though these in vitro results did not align with our in vivo data, the system provides an opportunity to define potential energy sources used by GMPs when mitochondrial pyruvate is not used.

**Figure 7.**
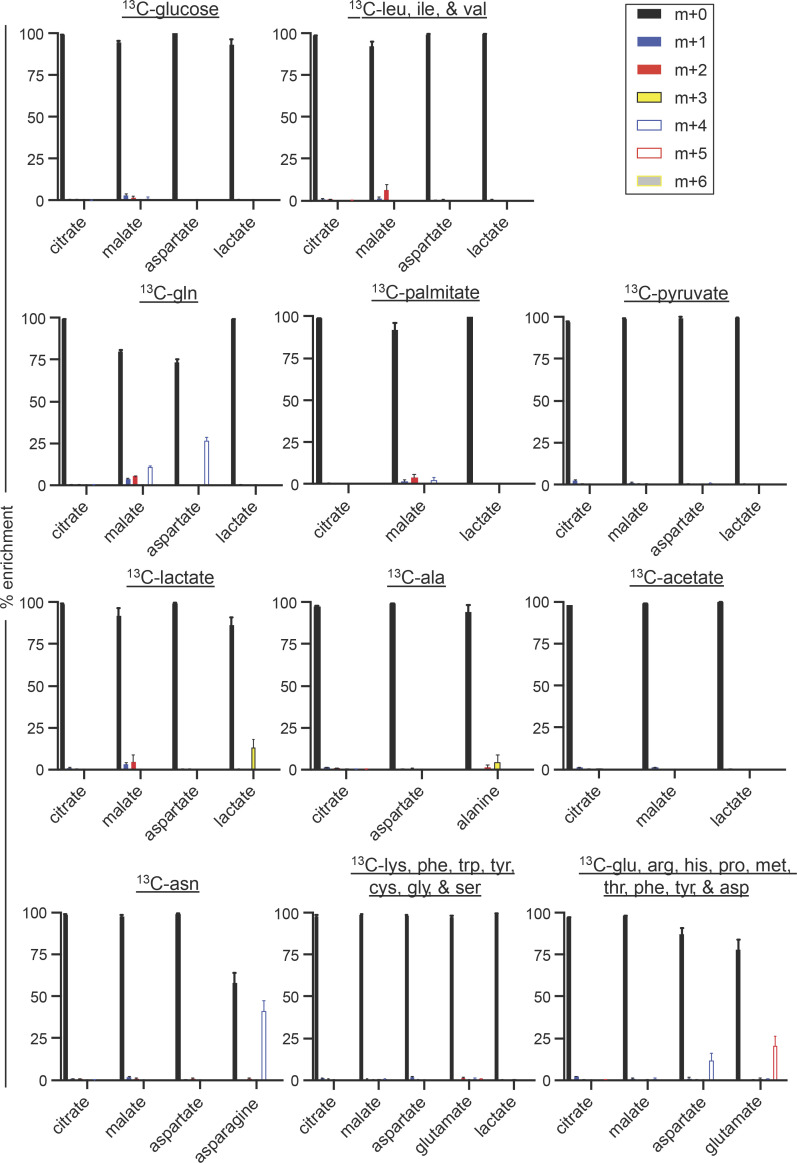
**GMPs use glutamine in vitro to generate TCA cycle intermediates.** LC/MS analysis of ^13^C incorporation into TCA cycle intermediates following a 24-h culture of WT GMPs with uniformly ^13^C-labeled glucose, BCAAs (leucine [leu]/isoleucine [ile]/valine [val]), glutamine (gln), palmitate, pyruvate, lactate, alanine (ala), acetate, asparagine (asn), lysine (lys)/phenylalanine (phe)/tryptophan (trp)/tyrosine (tyr)/cysteine (cys)/glycine (gly)/serine (ser), or glutamate (glu)/arginine (arg)/histidine (his)/proline (pro)/methionine (met)/threonine (thr)/phenylalanine (phe)/tyrosine (tyr)/aspartate (asp). Labeling data were corrected for natural-abundance ^13^C. Mean values ± SEM are shown for three to five replicates from two independent experiments for each ^13^C-labeled carbon source. Cells were pooled from five mice per each carbon source for each experiment.

To define the alternate carbon sources GMPs use in place of pyruvate, we performed stable isotope ^13^C tracing experiments. WT GMPs were cultured in media containing different ^13^C carbon sources for 24 h, and liquid chromatography/mass spectrometry (LC/MS) was used to analyze mass shifts in TCA cycle intermediates. ^13^C-glucose, which yields pyruvate through glycolysis, did not detectably contribute to the TCA cycle of in vitro GMPs (Fig. 7), consistent with our observations that UK5099 does not affect these cultures. We then traced ^13^C-labeled leucine, isoleucine, and valine to determine if GMPs utilize BCAAs in the TCA cycle given the upregulation of *Bcat1* in both recent and prolonged *Mpc2* KO GMPs relative to WT (Fig. 5, A–C). Labeling of TCA cycle intermediates was not observed (Fig. 7). With the upregulation of *Glul* in *Mpc2* recent KO GMPs relative to WT (Fig. 5 A), we also wanted to examine the usage of glutamine. Additionally, in a mouse model of colon cancer, *Mpc1* ablation in intestinal stem cells leads to the preferential use of the long-chain fatty acid palmitate in intestinal crypts, while adenomas utilize glutamine (Bensard et al., 2020). To determine if GMPs could be utilizing either of these carbon sources, we followed ^13^C-labeled glutamine and palmitate. While palmitate did not detectably contribute to the TCA cycle, carbons from glutamine did contribute to the intermediates malate and aspartate (Fig. 7). No contributions were observed from extracellular pyruvate directly or from lactate or alanine, which are also sources of pyruvate (Fig. 7). Additionally, no labeling of TCA cycle intermediates was observed following culture with ^13^C-acetate or the remaining amino acids (Fig. 7). These data indicate that myeloid progenitors use glutamine to drive the TCA cycle when not using pyruvate, at least in vitro.

### Mature myeloid cells fail to fully recover after deletion of both *Mpc2* and *Gls*

**Figure 8.**
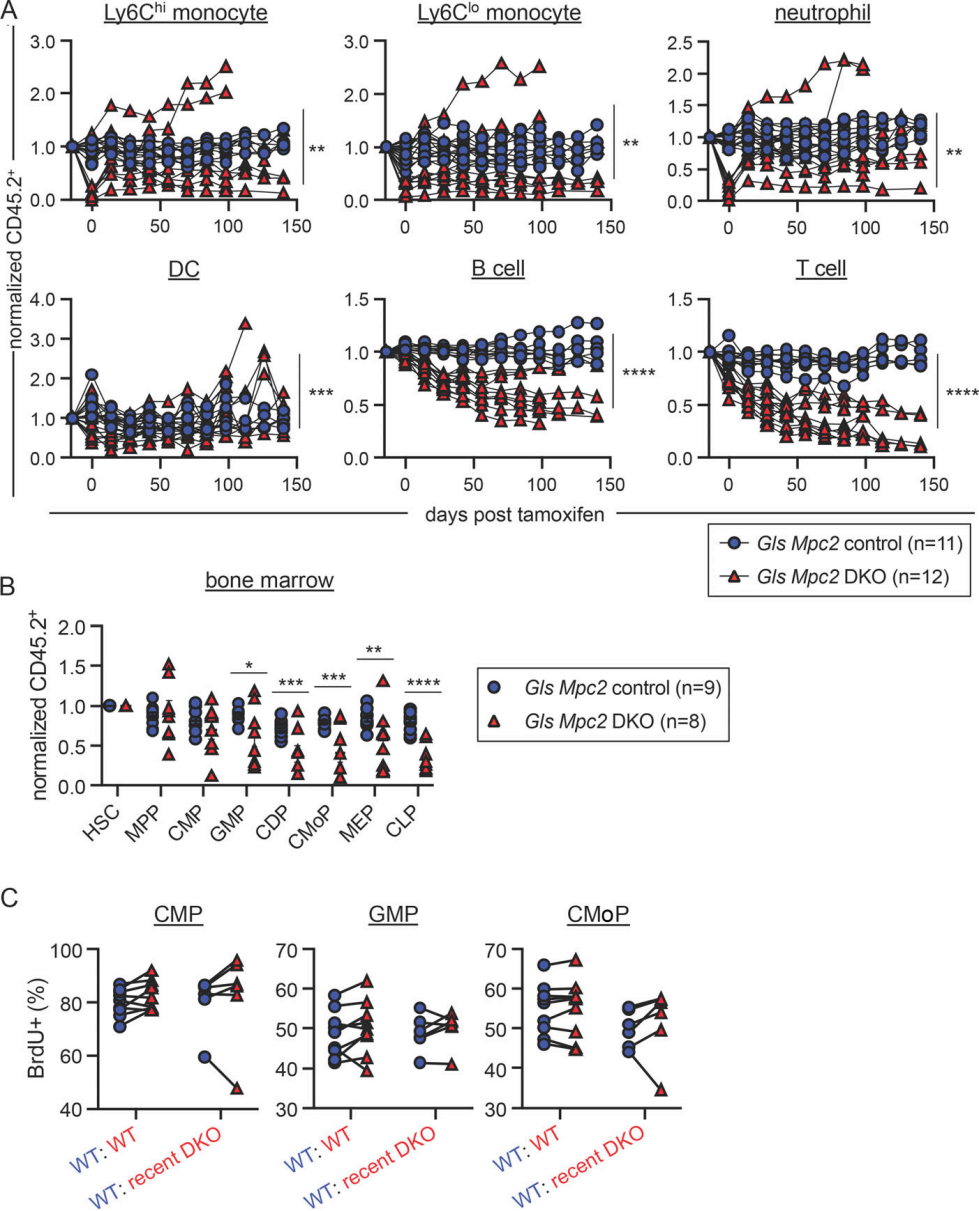
**Mature myeloid cells fail to fully recover after deletion of both *Mpc2* and *Gls*. (A)** Peripheral blood CD45.2 chimerism of mature cells was assessed every 2 wk. Values are normalized to pre-tamoxifen chimerism of each cell type. Each line represents longitudinal analysis of individual mice with a symbol at each time point measured. Data are pooled from two independent experiments. **, P < 0.01; ***, P < 0.001; ****, P < 0.0001 by paired two-way ANOVA with post-hoc Tukey’s multiple comparisons test. **(B)** CD45.2 chimerism of bone marrow progenitors normalized to HSC chimerism immediately following 2 wk of tamoxifen administration. Each symbol represents an individual mouse. Mean values ± SEM are shown. Data are pooled from two independent experiments. *, P < 0.05; **, P < 0.01; ***, P < 0.001; ****, P < 0.0001 by two-way ANOVA with post-hoc Tukey’s multiple comparisons test. **(C)** BrdU incorporation was measured in both CD45.1^+^ (WT) and CD45.2^+^ (WT or recent deletion of *Gls* and *Mpc2*) CMPs, GMPs, and CMoPs following a 1-h pulse of BrdU. Each line connects CD45.1^+^ and CD45.2^+^ cells within the same mouse (*n* = 9 for WT chimeras and *n* = 6 for recent DKO chimeras). Mice in which *Mpc2* was <90% reduced relative to WT controls were excluded. Data are pooled from two independent experiments. P values >0.05 by paired two-way ANOVA with post-hoc Sidak’s multiple comparisons test are not depicted.

**Figure S5.**
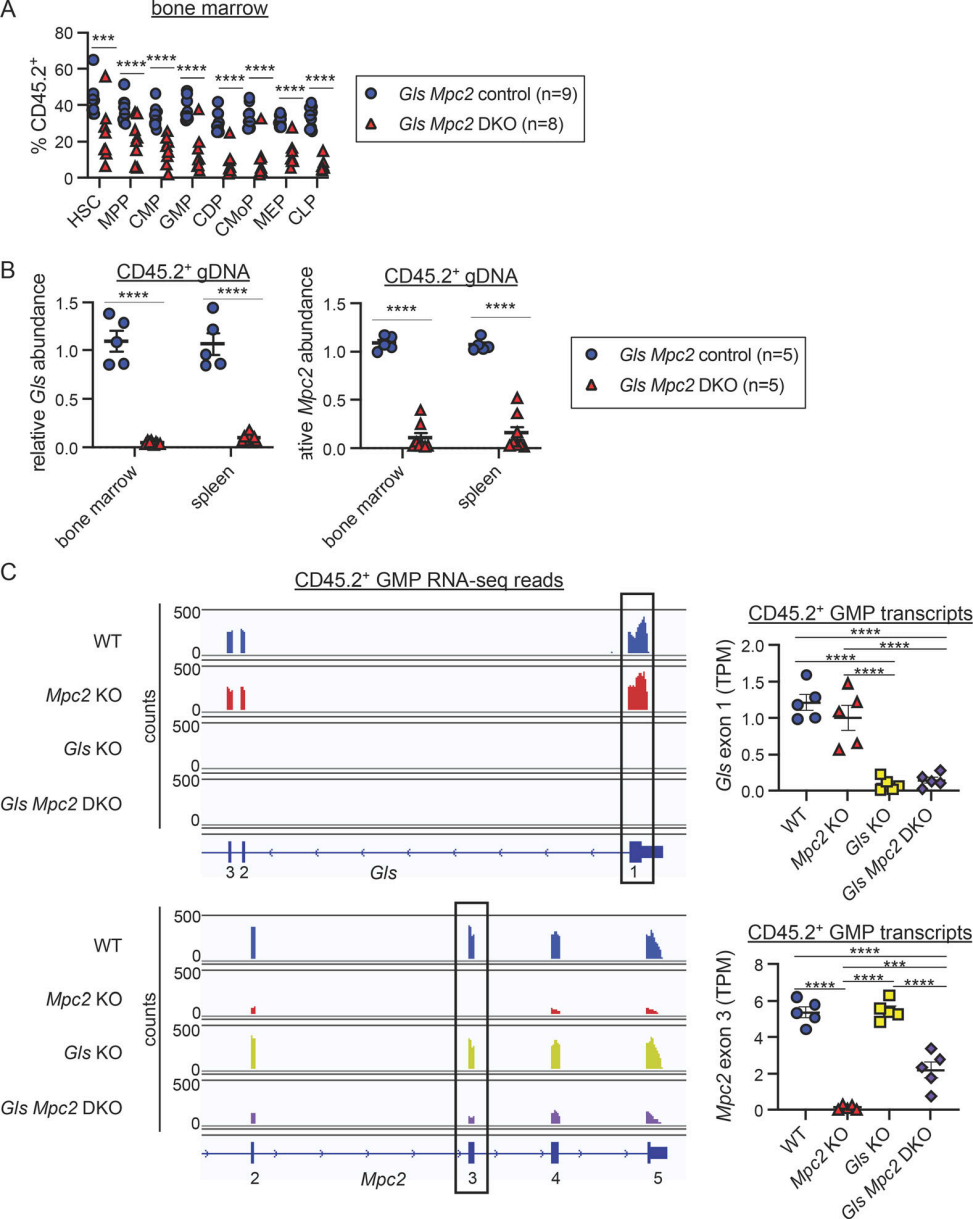
**Quantification of efficiency of *Gls* and *Mpc2* deletion. (A)** Raw chimerism frequencies for *Gls Mpc2* chimeras in Fig. 8 B. Each symbol represents an individual mouse. Mean values ± SEM are shown. Data are pooled from two independent experiments. ***, P < 0.001; ****, P < 0.0001 by two-way ANOVA with post-hoc Tukey’s multiple comparisons test. **(B)** Quantitative PCR analysis of genomic *Gls* and *Mpc2* deletion in recent *Gls Mpc2* KO chimeras. CD45.2^+^ cells from the bone marrow or spleen were sorted for genomic DNA extraction. DNA quantification within loxP sites was quantified relative to WT cells and GAPDH. Mean values ± SEM are shown. Data are pooled from two independent experiments. ****, P < 0.0001 by Student’s two-tailed *t* test. **(C)** RNA-seq reads across exons 1–3 of *Gls* (top) and 2–5 of *Mpc2* (bottom) of CD45.2^+^ GMPs from *ROSA26 CreER*^*+/−*^ (WT), *Mpc2* KO, *Gls* KO, or *Gls Mpc2* DKO chimeras about 10 wk after tamoxifen treatment. Exon 1 of *Gls* and exon 3 of *Mpc2* are floxed in the appropriate genotyped mice. One representative trace for each genotype is shown using Integrative Genomics Viewer (left). TPMs were quantified for the floxed exon of *Gls* (top right) or *Mpc2* (bottom right). TPMs were calculated from reads obtained using DEXSeq. Each data point represents a mouse (*n* = 5 for each genotype). Mean values ± SEM are shown. ***, P < 0.001; ****, P < 0.0001 by one-way ANOVA with post-hoc Tukey’s multiple comparisons test.

To determine if myeloid progenitors switch to glutaminolysis in vivo when mitochondrial pyruvate is unavailable, we crossed *Gls*^*fl/fl*^ mice to *Mpc2*^*fl/fl*^ mice (double KO [DKO]) and established mixed bone marrow chimeras. Myeloid cells from most DKO mutant chimeras experienced the initial sharp decline we observed in MPC2-deficient chimeras (Fig. 8 A). In these chimeras, myeloid cells recovered partially (Fig. 8 A), but not to the extent observed in myeloid cells that lacked only *Mpc2* (Fig. 3 A). Moreover, these results differ from those observed in cells lacking only *Gls*, which showed no competitive disadvantage to WT cells (Fig. 1 D and Fig. 3 C). Thus, the impaired recovery in DKO cells suggests that GMPs and CMoPs switch to glutaminolysis to recover from the loss of mitochondrial pyruvate. B and T cells were also progressively reduced upon deletion of both *Gls* and *Mpc2* (Fig. 8 A). DKO GMPs and CMoPs (Fig. 8 A; raw chimerism values in Fig. S5 A) were both reduced relative to *Mpc2* single KOs (Fig. 2 E). MEPs and CLPs were also reduced when both *Mpc2* and *Gls* were deleted (Fig. 8 B and Fig. S5 A). These data suggest other lineages can use either pyruvate or glutamine, whereas myeloid cells specifically require pyruvate but switch to glutaminolysis in its absence. Several DKO chimeras did not display myeloid cell loss (Fig. 8 A), likely due to inefficient deletion of *Mpc2* in these animals (Fig. S5, B and C). In contrast, *Gls* deletion was consistently efficient in DKO chimeras, as observed in genomic DNA from bone marrow cells and splenocytes after recent deletion (Fig. S5 B), and in RNA-seq of GMPs after prolonged deletion (Fig. S5 C).

To determine if the proliferation of myeloid progenitors was altered by the loss of both *Mpc2* and *Gls*, we performed a similar experiment as outlined in Fig. 4 A. Chimeras were injected with BrdU 1 h prior to sacrifice, and we assessed BrdU incorporation in both the WT CD45.1^+^ cells and control or recent DKO CD45.2^+^ cells within the same animals. Only chimeras in which *Mpc2* alleles were more than 90% deleted relative to WT controls were analyzed. The proliferation of WT and DKO CMPs, GMPs, and CMoPs was similar (Fig. 8 C). Similar levels of proliferation between DKO and WT cells are consistent with the minimal recovery of mature cells following the deletion of *Mpc2* and *Gls* (Fig. 8 A). These data demonstrate that glutamine is required for the proliferative burst of MPC2-deficient GMPs and CMoPs.

### Deletion of *Gls* delays emergency myelopoiesis

**Figure 9.**
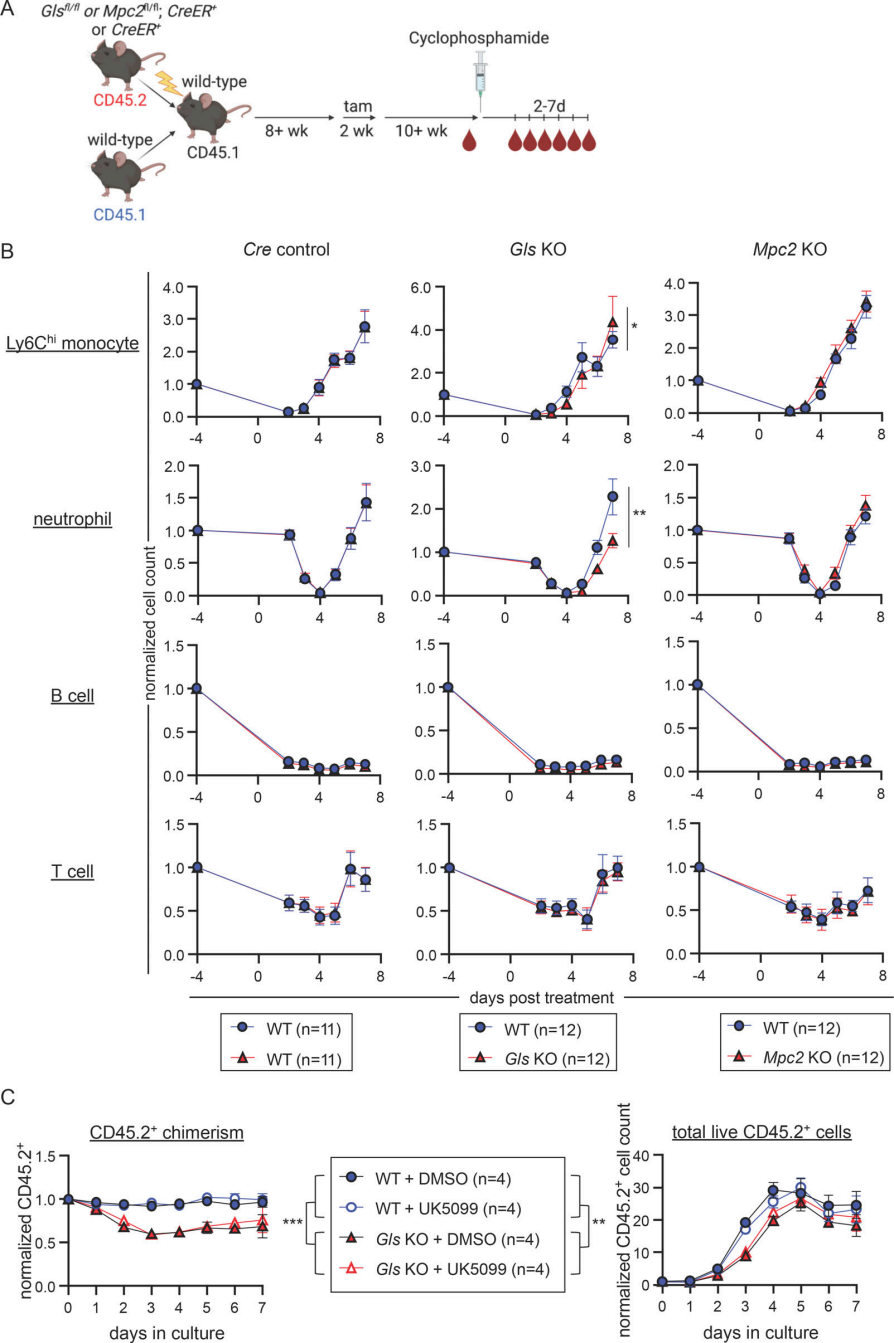
**Deletion of *Gls* delays emergency myelopoiesis. (A)** Schematic representation of cyclophosphamide experiment using *Gls* KO, *Mpc2* KO, and *Cre* only control mixed bone marrow chimeras. Chimeric mice were injected with cyclophosphamide and then were bled on days 2 through 7 after treatment. **(B)** Peripheral blood cell counts of both CD45.1^+^ (WT) and CD45.2^+^ (WT, *Gls* KO, or *Mpc2* KO) Ly6C^hi^ monocytes, neutrophils, B cells, and T cells from cyclophosphamide-treated chimeras. Values are normalized to pretreatment counts of each cell type. Data are pooled from two independent experiments. Mean values ± SEM are shown. *, P < 0.05; **, P < 0.01 by two-way ANOVA with post-hoc Tukey’s multiple comparisons test. **(C)** GMPs from WT or *Gls* KO chimeras were cultured with DMSO or 10 μM UK5099 in the presence of IL-3 and IL-6 for 7 d. On each day of the culture, cells were harvested and CD45.2 chimerism (left) and live cell counts (right) were obtained. Chimerism is normalized to the chimerism of the sorted GMPs. Cell counts are normalized to the total number of CD45.2^+^ cells plated on day 0. Data are pooled from two independent experiments with a total of 14 replicates from four mice per group. Mean values ± SEM are shown. **, P < 0.01; ***, P < 0.001 by two-way ANOVA with post-hoc Tukey’s multiple comparisons test.

We next performed experiments to determine if glutaminolysis is only utilized in the absence of mitochondrial pyruvate or if it is also important in other more physiological settings of emergency myelopoiesis, such as that induced by cyclophosphamide, a chemotherapeutic drug. After cyclophosphamide treatment, mature myeloid cells are rapidly depleted and then recover through emergency myelopoiesis (Jacquelin et al., 2013; Park et al., 2012; Duggan et al., 2017). Control, *Gls* KO, and *Mpc2* KO chimeras were injected with cyclophosphamide, and mature cells were measured in the periphery for up to 7 d after treatment (Fig. 9 A). We then compared the mature cell recovery in WT CD45.1^+^ cells with control, *Mpc2* KO, or *Gls* KO CD45.2^+^ cells within the same animal. We observed a delayed recovery by GLS-deficient Ly6C^hi^ monocytes and neutrophils relative to WT controls (Fig. 9 B). MPC2-deficient Ly6C^hi^ monocytes and neutrophils demonstrated a similar recovery to their WT counterparts. B and T cells were also reduced following cyclophosphamide treatment, but their recovery was not affected by *Gls* or *Mpc2* deletion (Fig. 9 B). The involvement of glutaminolysis in emergency myelopoiesis was also examined in vitro. GMPs were sorted from WT or *Gls* KO chimeras and cultured with SCF, GM-CSF, IL-3, and IL-6 to induce emergency myelopoiesis (Lantz et al., 1998; Weber et al., 2015; Zhan et al., 1998; Metcalf, 1980; Metcalf et al., 1986; Walker et al., 1985; Walker et al., 2008; Cutler et al., 1985; Ihle et al., 1983; Kindler et al., 1986; Metcalf et al., 1987; Romani et al., 1996; Pojda et al., 1992; Peters et al., 1997; Bernad et al., 1994; Patchen et al., 1991). Cells were additionally treated with DMSO or UK5099 to examine the role of pyruvate in emergency myelopoiesis. While UK5099 did not alter CD45.2 chimerism or cell counts in either WT or GLS-deficient cells, there was a significant decrease in GLS-deficient cells relative to WT that slightly recovered over time (Fig. 9 C). These data demonstrate glutaminolysis promotes emergency but not steady-state myelopoiesis (Fig. 1 D and Fig. 3 C).

**Figure 10.**
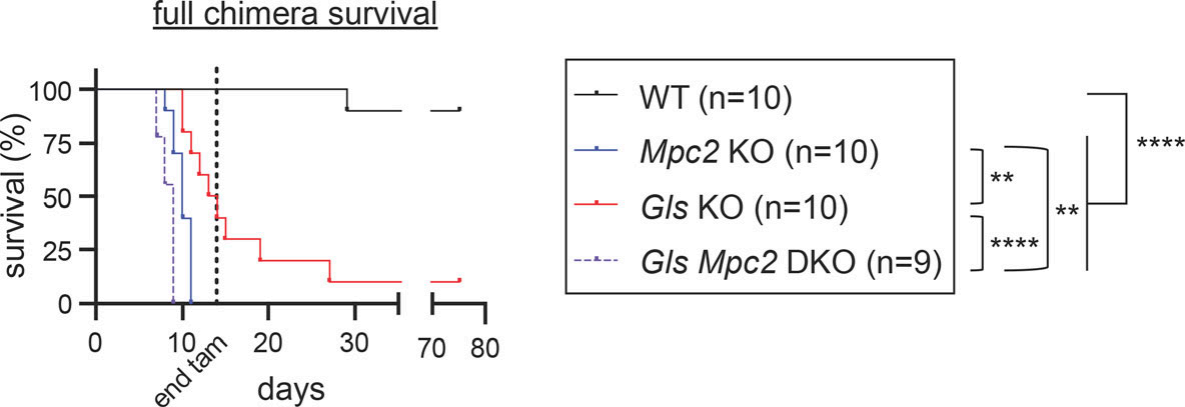
**Full chimeras do not survive deletion of *Mpc2* and/or *Gls*.** Full chimeras were generated with bone marrow from WT (*ROSA26 CreER*^*+/−*^), *Mpc2* KO (*Mpc2*^*fl/fl*^*; ROSA26 CreER*^*+/−*^), *Gls* KO (*Gls*^*fl/fl*^*; ROSA26 CreER*^*+/−*^), or *Gls Mpc2* DKO (*Gls*^*fl/fl*^ *Mpc2*^*fl/fl*^*; ROSA26 CreER*^*+/−*^) donors. Chimeras were provided tamoxifen chow for 14 d, and severe moribundity and survival were monitored over time. The single WT mouse that died was found in a flooded cage, suggesting an unrelated death. Data are pooled from two independent experiments. **, P < 0.01; ****, P < 0.0001 by log-rank test.

To further test the importance of *Mpc2* and/or *Gls* on steady-state and emergency myelopoiesis, we generated non-competitive chimeras with WT, *Mpc2* KO, *Gls* KO, or *Gls Mpc2* DKO donor bone marrow. Mice were provided tamoxifen chow to induce deletion and were monitored for signs of moribundity and survival. Severe neutropenia can cause mortality due to the inability to maintain microbial homeostasis at barrier surfaces. All *Gls Mpc2* DKO chimeras showed severe signs of moribundity warranting euthanasia or died between days 7 and 9 of tamoxifen administration (Fig. 10). *Mpc2* KO chimeras survived slightly longer, with animals persisting between 8 and 10 d. These data provide additional support for the importance for MPC2 in steady-state myelopoiesis. Unexpectedly, *Gls* KO chimeras also started dying at day 10, with just one mouse surviving at least 75 d following tamoxifen administration (Fig. 10). These data suggest that either emergency myelopoiesis is engaged relatively frequently even without intentional acute insults or that GLS is potentially required for hematopoietic effector functions that we did not test here.

## Discussion

Hematopoietic stem and progenitor cells maintain homeostasis by matching blood lineage replenishment to the rate of mature cell loss. A failure to maintain homeostasis can lead to myeloproliferative disorders and cancer, lymphopenia and susceptibility to infection, and anemia from reduced RBCs. To maintain homeostasis, stem and progenitor cells must continuously replenish mature cells, whether due to normal turnover of downstream lineages or from a significant loss of cells due to acute insults. In these two examples, the rates of replenishment required to maintain or restore homeostasis are markedly different. The extreme loss of mature cells necessitates the rapid proliferation and differentiation of progenitors (Bhattacharya et al., 2006; Cheshier et al., 2007). However, during a steady state, distinct pathways are presumably engaged to maintain but not overproduce cells. Our results here provide support for this concept, as mitochondrial pyruvate metabolism and glutaminolysis are toggled during steady-state and emergency myelopoiesis. Ensuring that only affected lineages are expanded and restored adds an additional level of specificity and complexity to this problem.

Under steady-state conditions, a progressive switch from glycolysis to oxidative phosphorylation promotes ATP production to support transit amplification (Takubo et al., 2013; Yu et al., 2013; Wang et al., 2014; Maryanovich et al., 2015; Ito and Suda, 2014; Nakamura-Ishizu et al., 2020; Simsek et al., 2010; Suda et al., 2011; Ho et al., 2017). Here, we demonstrated that the genetic ablation of pyruvate import into mitochondria sharply reduced mature myeloid cells, but these cells recovered rapidly due to a transient proliferative burst specifically by myeloid progenitors. Interestingly, this burst was observed in GMPs and CMoPs, but not in upstream CMPs. Perhaps, earlier progenitors developmentally closer to HSCs have not fully switched to oxidative phosphorylation and can continue to rely on glycolysis for energy production. The recovery of MPC2-deficient myeloid cells occurred in competition with WT cells, demonstrating that this proliferative burst was intrinsic to cells devoid of mitochondrial pyruvate import. The burst was lineage specific and only transient. Thus, MPC2-deficient myeloid cells did not continue to outcompete their WT competitors at later time points, which otherwise would have led to a myeloproliferative disorder. Moreover, an accompanying burst in multipotent and lymphoid progenitors, which might have led to an overproduction of irrelevant lineages, was not observed. These findings are consistent with prior studies showing relatively stable numbers of circulating mature cells despite marked changes in nutrient availability, altered by circadian rhythms and diet (Pickel and Sung, 2020; Potter et al., 2016; Schlierf and Dorow, 1973). These data further highlight the precise control progenitors have in maintaining homeostasis.

While the proliferative burst in progenitors promotes myeloid homeostasis after *Mpc2* ablation, stopping this burst following myeloid recovery would seem equally as important. One possible mechanism is that MPC2-deficient progenitors briefly switch to lactate production due to increased cytosolic pyruvate. This has been shown to occur in tumors as part of the Warburg effect, leading to the regeneration of nicotinamide adenine dinucleotide (NAD^+^), which in turn enables repeated cycles of glycolysis and biomass production to enable rapid proliferation (DeBerardinis et al., 2008; Vander Heiden et al., 2009; Warburg, 1925). Yet once MPC2-deficient progenitors switch to glutaminolysis, feedback mechanisms such as citrate-mediated inhibition of phosphofructokinase activity (Garland et al., 1963; Poorman et al., 1984) might shut down this increased glycolysis and normalize the proliferative rate. These changes are difficult to directly observe in vitro, as demonstrated by our experiments in which mitochondrial pyruvate was minimally utilized even by untreated WT myeloid progenitors. Yet consistent with this possible mechanism, deletion of lactate dehydrogenase A permanently impairs proliferation of hematopoietic progenitors in vivo, as does loss of the M2 isoform of pyruvate kinase (PKM2), which limits the initial formation of pyruvate (Wang et al., 2014).

When we ablated both *Gls* and *Mpc2*, the recovery of mature myeloid cells was significantly impaired, even though deletion of *Gls* alone had no discernable effect. This suggests that glutaminolysis is functionally required following acute loss of myeloid cells and/or for rapid progenitor proliferation but not steady-state myelopoiesis. Reciprocally, MPC2 is required for steady-state but not emergency myelopoiesis. Relevant to this point, many malignancies engage Warburg metabolism, which promotes conversion of pyruvate to lactate rather than oxidation in the mitochondria. Indeed, the removal of glutamine from cultures, knocking down glutamine transporters, and pharmacologically inhibiting GLS in acute myeloid leukemia reduce leukemic cell proliferation while increasing apoptosis (Gallipoli et al., 2018; Jacque et al., 2015; Willems et al., 2013). Our genetic data here demonstrate that glutaminolysis inhibition does not impact normal progenitors under steady-state conditions, suggesting that such treatments are worth exploring for the treatment of disorders such as acute myeloid leukemia.

While our data demonstrate an important role for glutamine in the absence of mitochondrial pyruvate in myeloid cells, the ablation of both *Gls* and *Mpc2* did not eliminate these populations and a slight recovery from the initial decline was observed. Additionally, in cyclophosphamide-induced myeloablation experiments, GLS-deficient myeloid cells had a delayed recovery relative to controls but still did return to pretreatment levels. Thus, there might be additional carbon sources that promote recovery. For example, while MPC1 deficiency in mice leads to lethality around embryonic day 13.5, administering pregnant dams a ketogenic diet restores embryonic development (Vanderperre et al., 2016). Additionally, ablation of *Mpc1* in the intestinal epithelium of mice leads to increased palmitate oxidation relative to WT controls in ex vivo intestinal crypts (Bensard et al., 2020). Similarly, although myeloid progenitors primarily switch to glutaminolysis in the absence of mitochondrial pyruvate, we have previously observed that MPC2-deficient neutrophils can be rescued by retroviral transduction with a long-chain fatty acid transporter (Lam et al., 2016; Schaffer and Lodish, 1994). Although we did not observe contributions from palmitate in vitro, preliminary experiments showed a selection against cells that had deleted both *Cpt2* and *Mpc2* in DKO chimeras. These data suggest a possible role for long-chain fatty acid oxidation to complement MPC2 deficiency in vivo. Additionally, while we did not trace carbons from BCAAs to TCA cycle intermediates in vitro, RNA-seq revealed an upregulation of *Bcat1* in MPC2-deficient GMPs relative to WT, suggesting BCAAs may also be utilized.

When we depleted mature myeloid cells with cyclophosphamide in chimeras to observe the role of metabolic pathways in emergency myelopoiesis, GLS-deficient myeloid cells had a delayed recovery relative to controls. Given that glutaminolysis is dispensable for homeostatic myelopoiesis, these data suggest that different metabolic pathways are required to maintain cells at a steady state and to promote recovery following an acute insult. This difference in metabolic pathways utilized could be connected to factors that differ between steady-state and emergency myelopoiesis, such as cytokines. GM-CSF, G-CSF, and M-CSF promote survival, proliferation, and myeloid differentiation. Following systemic infection, which commonly induces emergency hematopoiesis or in the context of myeloproliferative disorders, the concentrations of these cytokines in the serum are up to 100-fold higher than those found in the steady state (Hamilton, 2008; Kawakami et al., 1990; Selig and Nothdurft, 1995; Watari et al., 1989). Thus, the in vitro experiments we performed with progenitors may have been more representative of emergency myelopoiesis, thereby explaining why we did not observe any functional contribution of mitochondrial pyruvate metabolism. With the addition of IL-3 and IL-6 to cultures to further induce emergency myelopoiesis, we again did not observe a phenotype with MPC inhibition. We did, however, see reduced proliferation of GLS-deficient cells relative to controls. Differences in the levels of these cytokines might mediate metabolic switches during steady-state and emergency myelopoiesis. Similarly, proliferation-terminating cytokines, such as transforming growth factor β1 (TGFβ1), might act through metabolic pathways to enforce relative quiescence (Hérault et al., 2017).

Nonetheless, the proliferation of myeloid progenitors triggered by the loss of MPC2 was initiated cell-intrinsically rather than by feedback from loss of mature cells. These data suggest the ability of progenitors to adjust in anticipation of the loss of their mature progeny. In the example we provide here, this adaptation is triggered by a metabolic switch that more dramatically impacts mature cells than the upstream progenitors. In this setting, glutaminolysis is again required for rapid recovery. Together, the data suggest that pyruvate metabolism and glutaminolysis serve as general metabolic toggles to switch between steady-state and emergency myelopoiesis.

## Materials and methods

### Mice

All animal procedures used in this study were approved by the Animal Care and Use Committees at Washington University in St. Louis and the University of Arizona. C57BL6/N and B6.Ly5.2 (CD45.1) mice were obtained from Charles River Laboratories. β-Actin GFP transgenic mice were obtained from The Jackson Laboratory (stock number 006567). *Mpc2*^*fl/fl*^ mice were kindly provided by B.N. Finck (McCommis et al., 2015; currently available from The Jackson Laboratory, stock number 032118). *Cpt2*^*fl/fl*^ mice were kindly provided by M.J. Wolfgang (Lee et al., 2015). *Gls*^*fl/fl*^ mice were obtained from The Jackson Laboratory (stock number 017894; Mingote et al., 2016). All floxed mouse lines were crossed to *ROSA26 CreER* mice obtained from The Jackson Laboratory (stock number 008463). Littermates were used when possible.

### Mixed bone marrow chimeras

To generate mixed bone marrow chimeras, bone marrow cells from a CD45.2 donor mouse carrying floxed alleles were mixed 1:1 or 1:9 with bone marrow cells from B6.Ly5.2 CD45.1 mice (or β-actin GFP transgenic mice for *Gls* chimeras). Full chimeras were generated using only bone marrow cells from CD45.2 donor mice. 10 × 10^6^ total mixed bone marrow cells were injected retro-orbitally into 800 cGy-irradiated B6.Ly5.2 CD45.1 mice. Recipient mice were administered Sulfatrim (40 mg/ml sulfamethoxazole and 8 mg/ml trimethoprim) via drinking water for 2 wk. Chimeras were rested for at least 8 wk to allow reconstitution. Peripheral blood chimerism was assessed through tail venipuncture prior to tamoxifen administration. To induce deletion, tamoxifen was administered in their diet (400 mg tamoxifen citrate/kg diet; Envigo) for 2 wk.

For *Mpc2* retroviral chimeras, bone marrow cells were c-kit–enriched using CD117 microbeads (Miltenyi Biotec), and 50,000–100,000 cells were cultured in a 96-well plate in DMEM/F12 supplemented with 10% FBS, HEPES, non-essential amino acids, sodium pyruvate, glutamine, 50 μM β-mercaptoethanol (Gibco), 50 ng/ml SCF (Peprotech), and 50 ng/ml thrombopoietin (Peprotech) at 37°C with 5% CO_2_ (Lam et al., 2016). The following day, 5 μl of *Mpc2* retrovirus was added to each well. A day after infection, cells were mixed 1:1 with WT CD45.1^+^ cells (similarly cultured but not infected) and transplanted as described above. For retrovirus production, *Mpc2* constructs were cloned into a murine stem cell virus–IRES plasmid. Lenti-X 293Ts (Clonentech) were transfected at about 60% confluency in a 10 cm^2^ tissue culture dish using 30 μl FuGene HD (Promega), 1.75 μg pMD2.G vector, 3.25 μg pBS CMV-gag-pol (Addgene), and 5 μg *Mpc2* retroviral vector. 4–6 h after transfection, the medium was changed. At both 48 and 72 h later, the supernatant was collected and filtered through 0.45 μm syringe filters. 3 ml of 1× PBS/25% polyethylene glycol 8000 (Sigma-Aldrich) was mixed with 12 ml of the harvested viral supernatant and then incubated at 4°C. The following day, the mixture was spun down at 3,000 *g* for 20 min. The supernatant was removed, and 100 μl of PBS was used to resuspend the pellet. Aliquots were prepared and then stored at −80°C until the time of use.

### Mouse tissue processing

Spleens were harvested and dissociated using frosted glass microscope slides. Femurs, tibiae, and pelvic bones were isolated and crushed with a mortar and pestle. RBCs were lysed using a 0.15 M NH_4_Cl, 10 mM KHCO_3_, 0.1 mM EDTA, pH 7.2 solution (ACK). Non-cellular debris was removed by gradient centrifugation for 10 min at 2,000 *g* using Histopaque 1119 (Sigma-Aldrich). Interface cells were collected, washed, and then filtered through a 70 μm nylon mesh prior to antibody staining or downstream applications. Peripheral blood was collected in 10 mM EDTA/PBS via tail venipuncture of warmed mice. RBCs were lysed with ACK prior to antibody staining. For platelets, immediately following bleeding and prior to ACK lysis, 2% dextran sulfate/PBS was added to blood samples. Samples were centrifuged at 200 *g* for 20 min at room temperature. The supernatant was transferred to a new tube and centrifuged at 1,100 *g* for 10 min at room temperature to pellet platelets.

### Flow cytometry

Single-cell suspensions were prepared from spleen, bone marrow, or peripheral blood. Cells were resuspended in PBS with 5% adult bovine serum and 2 mM EDTA prior to staining. The following antibodies were purchased from BioLegend: CD3 (clone 17A2)—PE/Dazzle 594, Brilliant Violet 510, PerCP/Cy5.5; B220 (RA3-6B2)—Alexa Fluor 700, APC, Brilliant Violet 421, FITC; I-A/I-E (M5/114.15.2)—Biotin, Brilliant Violet 711; Ly-6C (HK1.4)—Brilliant Violet 510, FITC; Ly-6G (1A8)—Brilliant Violet 785, FITC, PE; CD45.1 (A20)—APC/Cyanine 7, Brilliant Violet 605, PE/Cy7; CD45.2 (104)—APC/Cy7, Brilliant Violet 421, Brilliant Violet 510; NK-1.1 (PK136)—PE; IgD (11-26c.2a)—Brilliant Violet 605; CD90.1 (OX-7)—FITC; CD115 (AFS98)—Brilliant Violet 711; CD127 (A7R34)—Biotin; CD138 (281-2)—Brilliant Violet 650, Brilliant Violet 510; CD150 (TC15-12F12.2)—Brilliant Violet 421, Alexa Fluor 647; IgM (RMM-1)—APC; Ly-6A/E (E13-161.7)—PE; Ly-6G/Ly-6C (RB6-8C5)—APC; Streptavidin—APC/Cy7, Brilliant Violet 650, Brilliant Violet 711; CD61 (2C9.G2 [HMβ3-1])—PE; CD8 (53-6.7)—Alexa Fluor 700; CD11b (M1/70)—Alexa Fluor 488, PerCP/Cy5.5; CD11c (N418)—APC/Cyanine7, Biotin; CD16/32 (93)—PerCP/Cyanine5.5; CD34 (MEC14.7)—Brilliant Violet 421. The following antibodies were purchased from eBioscience: CD115 (AFS98)—Biotin; Ly-6G/Gr-1 (RB6-8C5)—PE-Cy5; CD4 (GK1.5)—PE-Cy5; CD8 (53-6.7)—PE-Cy5; CD11b (M1/70)—PE-Cy5; CD27 (LG.7F9)—APC; CD34 (RAM34)—FITC. The following antibodies were purchased from BD: CD11b (M1/70)—BUV395, BUV661, PE-Cy7; CD4 (RM4-5)—PE-Cy7; CD8 (53-6.7)—BUV 805; CD135 (A2F10.1)—PECF594; Streptavidin—BV421, BV605; CD3 (clone 17A2)—PE; CD27 (LG.3A10)—BUV805; CD34 (RAM34)—BV786. The following antibodies were purchased from Invitrogen: CD11c (N418)—PerCP-Cyanine5.5; CD115 (AFS98)—APC; IgM (II/41)—PerCP-eFluor 710, FITC; CD117 (2B8)—PE-Cyanine7; TER-119 (TER-119)—PE-Cyanine7; CD4 (RM4-5)—Pacific Orange; CD16/32 (93)—APC; and CD34 (RAM34)—Alexa Fluor 700. DAPI and propidium iodide were purchased from Sigma-Aldrich. Cells were analyzed on a BD LSR II, BD Fortessa, BD Fortessa X-20, or Cytek Aurora. All fluorescence-activated cell sorting was performed on a BD FACS Aria II or III. Data were analyzed using FlowJo software (FlowJo Enterprise).

### Quantitative PCR analysis

To quantify the deletion of *Cpt2*, *Gls*, and *Mpc2*, 50,000–150,000 CD45.2^+^ cells were sorted from the spleen, bone marrow, or peripheral blood of chimeras into PBS with 5% adult bovine serum. DNA was isolated using the Genomic DNA Mini Kit (IBI Scientific). Quantitative PCR was then used to determine the extent of deletion. Normalization control primers amplified a region of GAPDH (forward primer: 5′-AAC​TTT​GGC​ATT​GTG​GAA​GG-3′ and reverse primer 5′-GGA​TGC​AGG​GAT​GAT​GTT​CT-3′). Primers for *Cpt2*, *Gls*, and *Mpc2* amplified a region within the loxP sites. The *Cpt2* forward primer was 5′-GAT​GGC​TGA​GTG​CTC​CAA​AT-3′, and the reverse was 5′-GCC​AGA​CCC​AAG​GTG​TTC​T-3′. The *Gls* forward primer was 5′-ATC​TCC​TTG​CCC​TCG​CTG​T-3′, and the reverse was 5′-CGC​CCT​CGG​AGA​TCC​TAC-3′. The *Mpc2* forward primer was 5′-CCC​CAC​CGT​GTC​TTA​ATG​TC-3′, and the reverse was 5′-CAC​AGT​GGA​CTG​AGC​TGT​GC-3′. PCR reactions were performed in 20 μl containing SYBR Green PCR Master Mix (Applied Biosystems) and 10 μM of each primer. Reactions were performed in a StepOnePlus realtime qPCR machine (Applied Biosystems) with 40 cycles of the following program: 94°C, 30 s; 59.1°C, 30 s; 72°C, 30 s; followed by a melt-curve analysis. Quantification was calculated using normalized ΔC_t_ values. An average from WT samples was used to estimate a value of 1.

### BrdU pulse and pulse-chase experiments

For BrdU pulse experiments, mice were injected with 1 mg BrdU intraperitoneally 1 h prior to sacrifice. For BrdU pulse-chase experiments, mice were provided with 0.8 mg/ml BrdU in their drinking water, replaced daily for 7 d. At the end of the 7 d, mice were returned to normal drinking water and were bled that day and the following 3 d. Cells were then processed and stained for BrdU incorporation with the FITC BrdU Flow Kit (BD) per the manufacturer’s instructions.

### RNA-seq

Single-cell suspensions of bone marrow cells were generated as per the tissue processing section. CD27^+^ cells were enriched using APC microbeads (Miltenyi Biotec). CD45.2^+^ GMPs were sorted from WT (*ROSA26 CreER*^*+/−*^), *Mpc2*, *Gls*, and *Gls Mpc2* KO chimeras at the end of tamoxifen treatment or about 10 wk after tamoxifen treatment into Buffer RA1 (Macherey-Nagel). RNA was isolated using the NucleoSpin RNA XS kit (Macherey-Nagel). Sequencing libraries were generated by Novogene using the SMARTseq V4 kit (Takara Bio). Paired-end 150 bp reads were acquired by Novogene using Illumina NovaSeq. For quantification of differences in gene expression, reads were mapped to Gencode’s M27 annotation files, and transcript abundances were calculated using Salmon (Patro et al., 2017). DESeq2 was used to quantify differentially expressed genes (Love et al., 2014). To analyze exon usage, fastq files were first aligned to the mm10 genome using HISAT2 (Kim et al., 2015). HISAT2 bam files were then visualized using Integrative Genomics Viewer (Thorvaldsdóttir et al., 2013) and used to quantify transcripts per kilobase million (TPM) for floxed exons via DEXSeq (Anders et al., 2012). RNA-seq data are available in the National Center for Biotechnology Information Gene Expression Omnibus (NCBI GEO; recent KO accession number GSE225578 and prolonged KO accession number GSE184548). Venn diagrams were generated at https://www.meta-chart.com/venn#/data.

### GMP cultures

Single-cell suspensions of bone marrow cells were generated as per the tissue processing section. C-kit^+^ cells were enriched using CD117 microbeads (Miltenyi Biotec). GMPs were sorted into RPMI containing 10% FBS, glutamine, and penicillin/streptomycin. 50,000–100,000 GMPs were cultured for pyruvate excretion analysis or ^13^C tracing experiments, while 1,000 GMPs were plated for 7-d differentiation analysis in RPMI containing 10% FBS, glutamine, penicillin/streptomycin, 10 ng/ml GM-CSF (PeproTech), and 10 ng/ml SCF (PeproTech). When C57BL6/N mice were used as the source of GMPs, DMSO or 10 μM UK5099 (Sigma-Aldrich) was added to the medium as vehicle control or to inhibit MPC. When GMPs were obtained from *Mpc2*^*fl/fl*^*; ROSA26 CreER*^*+/−*^ or *ROSA26 CreER*^*+/−*^ mice, 0.5 μM 4-OHT (Sigma-Aldrich) was added to the medium at the time of plating. On day 2 of culture, the medium was changed to fresh medium without 4-OHT. For emergency myelopoiesis experiments, GMPs were obtained from chimeras following 2 wk of tamoxifen administration. Emergency myelopoiesis was induced by adding 10 ng/ml IL-3 (Peprotech) and 10 ng/ml IL-6 (Peprotech) to the culture medium. Cells were incubated at 37°C with 5% CO_2_.

### Nutrient-uptake analysis

GMPs were sorted and cultured as described above. GMPs were collected on day 3 of culture, washed with PBS, replated in fresh media, and returned to the incubator for 48 h. The spent media was collected. The extraction solvent methanol/acetonitrile (ACN)/water (2:2:1) containing isotope-labeled internal standards ([U-^13^C_6_]glucose; [U-^13^C_3_]lactate; [U-^13^C_5_]glutamine; and [U-^13^C_5_]glutamate; Cambridge Isotope Laboratories) were spiked into media samples. Samples were vortexed for 30 s, incubated for 1 min in liquid nitrogen, and then sonicated for 10 min. Following a 1-h incubation at −20°C, the samples were centrifuged at 14,000 rpm for 10 min. The supernatant was analyzed by using an Agilent 1290 UHPLC system coupled to an Agilent 6540 quadruple time-of-flight mass spectrometer. The consumption rates (*x*) were normalized by cell growth over the experimental time period using the following algorithm (*N*_*0*_, starting cell number; *t*, incubation time; *DT*, doubling time; and *Y*, nutrient utilization).

$$Y=\int_{0}^{t}x\cdot N_{0}\cdot 2^{\frac{t}{DT}}\cdot dt$$

### Isotope labeling and metabolite extraction

GMPs were sorted and cultured as described above. Cells were collected on day 4 of culture, washed with PBS, and replated in the appropriate media (listed below) for 24 h. Following incubation, cells were collected, washed with PBS, washed with high-performance liquid chromatography (HPLC)–grade water, and quenched with HPLC-grade methanol. Pellets were dried using a SpeedVac. Dried cell pellets were extracted with 1 ml methanol/ACN/water (2:2:1). Samples were vortexed for 30 s, incubated for 1 min in liquid nitrogen, and then sonicated for 10 min. This process was repeated three times. After a 1-h incubation at −20°C, samples were centrifuged at 14,000 rpm for 10 min. The supernatant was collected and dried via SpeedVac and reconstituted in ACN/water (1:1) for LC/MS analysis.

All U-^13^C carbon sources were obtained from Cambridge Isotope Laboratories. For all ^13^C experiments, the base medium was RPMI containing 10% FBS, glutamine, penicillin/streptomycin, 10 ng/ml GM-CSF, and 10 ng/ml SCF, unless noted otherwise, and supplemented with ^13^C carbon sources. For glucose, glucose-free RPMI was used to make the base medium instead of standard RPMI and was supplemented with 11.1 mM ^13^C-glucose. For glutamine, glutamine-free RPMI was used, glutamine was left out of the base medium, and 4 mM ^13^C-glutamine was added. For palmitate, 100 μM ^13^C-palmitate-BSA and 100 μM oleate-BSA was added. For pyruvate, 100 μM ^13^C-pyruvate was supplemented. Concentrations used for all other ^13^C tracing experiments were as follows: 3 mM ^13^C-lactate, 400 μM ^13^C-alanine, 500 μM ^13^C-acetate, 430 μM ^13^C-asparagine, 382 μM ^13^C-leucine, 382 μM ^13^C-isoleucine, 171 μM ^13^C-valine, 220 μM ^13^C-lysine, 91 μM ^13^C-phenylalanine, 60 μM ^13^C-tryptophan, 110 μM ^13^C-tyrosine, 336 μM ^13^C-cysteine, 133 μM ^13^C-glycine, 286 μM ^13^C-serine, 136 μM ^13^C-glutamate, 1.15 mM ^13^C-arginine, 97 μM ^13^C-histidine, 174 μM ^13^C-proline, 101 μM ^13^C-methionine, 168 μM ^13^C-threonine, 91 μM ^13^C-phenylalanine, 110 μM ^13^C-tyrosine, and 150 μM ^13^C-aspartate.

### LC/MS analysis

Media samples and cell extracts were analyzed with Agilent 1290 UHPLC system coupled to an Agilent 6540 quadruple time-of-flight mass spectrometer. Polar metabolites were separated on a zic-pHILIC column (100 × 2.1 mm, 5 μm, polymer; Merck-Millipore) with a ZIC-pHILIC guard column (20 × 2.1 mm, 5 μm, polymer; Merck-Millipore). The column compartment temperature was 40°C. Mobile phase A was 95% water and 5% ACN with 20 mM ammonium bicarbonate, 0.1% ammonium hydroxide solution, and 2.5 μM medronic acid. The ammonium hydroxide solution was purchased as 25% ammonia in water (Honeywell). Mobile phase B was 95% ACN and 5% water with 2.5 μM medronic acid. Separation was carried out with a flow rate of 250 μl min^−1^ and the following linear gradients: 90% B from 0 to 1 min, 90–25% B from 1 to 14 min, 25% B from 14 to 15.5 min, and 25% B to 90% B from 15.5 to 18 min. The column was equilibrated with 90% B for 10.5 min at a flow rate of 400 μl min^−1^ and 1.5 min at a flow rate of 250 μl min^−1^. Mass spectrometry detection was accomplished in negative ionization mode with the following settings: gas temperature, 200°C; sheath gas temperature, 300°C; drying gas flow rate, 10 liters min^−1^; sheath gas flow rate, 12 liters min^−1^; nebulizer pressure, 44 psi; capillary voltage, 3,000 V; nozzle voltage, 2,000 V; fragmentor voltage, 100 V; skimmer voltage, 65 V; and scan rate, 1 spectrum s^−1^.

### Cyclophosphamide experiments

Mice were injected with 175 mg/kg cyclophosphamide (Sigma-Aldrich) in PBS intraperitoneally (Duggan et al., 2017). Prior to treatment and on days 2–7 after treatment, mice were bled as described above into dipotassium EDTA-coated tubes (BD). Blood samples were analyzed on an Element HT5 (Heska) to obtain white blood cell counts. Flow cytometry was used as described above to determine the frequency of each cell population. Cell frequencies were applied to white blood counts to obtain a cell count for each population of interest. Cell counts for each day were then normalized to pretreatment cell counts.

### Statistical analysis

All statistical analyses were conducted using GraphPad Prism and are detailed in each figure legend. Figures were created with BioRender.com.

### Online supplemental material

Fig. S1 shows the general flow cytometry gating strategy for cell type identification used throughout the article and confirms the deletion of *Cpt2* and *Gls*. Fig. S2 shows the raw chimerism values for *Cpt2* and *Gls* KO chimeras. Fig. S3 shows the flow cytometry gating strategy used for additional myeloid subsets, demonstrates *Mpc2* retroviral rescue of MPC2-deficient myeloid cells, confirms deletion of *Mpc2*, and includes raw chimerism values for *Mpc2* KO chimeras. Fig. S4 includes raw chimerism data for *Mpc2* KO chimera bleeds over time, BrdU pulse, and pulse-chase data for additional cell types, as well as the kinetics of mature myeloid cell loss and recovery in mixed bone marrow chimeras with 90% WT and 10% *Mpc2* KO cells. Fig. S5 shows raw chimerism values for *Gls Mpc2* DKO chimeras and the extent of deletion of *Gls* and *Mpc2* in these chimeras.

## Data Availability

RNA-seq data from Fig. 5 are available in the NCBI GEO. Data for recent and prolonged MPC2-deficient GMPs are under accession numbers GSE225578 and GSE184548, respectively. All other data are available in the article or upon reasonable request to the corresponding author.
